# Automated High-Throughput Characterization of Single Neurons by Means of Simplified Spiking Models

**DOI:** 10.1371/journal.pcbi.1004275

**Published:** 2015-06-17

**Authors:** Christian Pozzorini, Skander Mensi, Olivier Hagens, Richard Naud, Christof Koch, Wulfram Gerstner

**Affiliations:** 1 Laboratory of Computational Neuroscience (LCN), Brain Mind Institute, School of Computer and Communication Sciences and School of Life Sciences, École Polytechnique Fédérale de Lausanne, Lausanne, Switzerland; 2 Laboratory of Neural Microcircuitry (LNMC), Brain Mind Institute, School of Life Sciences, École Polytechnique Fédérale de Lausanne, Lausanne, Switzerland; 3 Department of Physics, University of Ottawa, Ottawa, Canada; 4 Allen Institute for Brain Science, Seattle, Washington, USA; The University of Texas at Austin, UNITED STATES

## Abstract

Single-neuron models are useful not only for studying the emergent properties of neural circuits in large-scale simulations, but also for extracting and summarizing in a principled way the information contained in electrophysiological recordings. Here we demonstrate that, using a convex optimization procedure we previously introduced, a Generalized Integrate-and-Fire model can be accurately fitted with a limited amount of data. The model is capable of predicting both the spiking activity and the subthreshold dynamics of different cell types, and can be used for online characterization of neuronal properties. A protocol is proposed that, combined with emergent technologies for automatic patch-clamp recordings, permits automated, *in vitro* high-throughput characterization of single neurons.

## Author Summary

Large-scale, high-throughput data acquisition is revolutionizing the field of neuroscience. Single-neuron electrophysiology is moving from the situation where a highly skilled experimentalist can patch a few cells per day, to a situation where robots will collect large amounts of data. To take advantage of this quantity of data, this technological advance requires a paradigm shift in the experimental design and analysis. Presently, most single-neuron experimental studies rely on old protocols—such as injections of steps and ramps of current—that rarely inform theoreticians and modelers interested in emergent properties of the brain. Here, we describe an efficient protocol for high-throughput *in vitro* electrophysiology as well as a set of mathematical tools that neuroscientists can use to directly translate experimental data into realistic spiking neuron models. The efficiency of the proposed method makes it suitable for high-throughput data analysis, allowing for the generation of a standardized database of realistic single-neuron models.

This is a *PLOS Computational Biology* Methods paper.

## Introduction

*In vitro* patch-clamping is the gold standard used to investigate the intrinsic electrophysiological properties of neurons, but remains labour intensive and requires a trained experimentalist with high technical skills. In the last years, several platforms have been developed that automatize electrophysiological recordings for ion-channel screening and drug discovery [1]. Most of the existing platforms are, however, designed to record from mammalian cell lines or oocytes in which ion-channels of interest are artificially expressed [2, 3]. In the near future, this technology is likely to be transferred to more complex setups, such as *in vitro* brain slices. High-throughput electrophysiology can be pushed forward with *in vivo* whole-cell patch-clamp recordings that are, at least partially, automatized [4]. With this technique, three to seven minutes are sufficient for a trained technician or a robot to automatically identify a cell and form a gigaohm seal of the same quality as achieved by an electrophysiologist [4]. This technological advance represents an important step towards high-throughput electrophysiology *in vivo* or on *in vitro* brain slices.

To make sense of the large amount of data that automated patch-clamp can produce, adequate computational tools and experimental protocols have to be developed. Traditional protocols for single-neuron characterization rely on current-clamp injections of stimuli (e.g., square current pulses, ramps of current) that are specifically designed to extract a small number of parameters (e.g., membrane time constant, firing threshold). While this is a valid approach, the input currents adopted in these experiments are artificial and strongly differ from the signals that single neurons process *in vivo*. Moreover, the choice of the parameters used for single-neuron characterization is arbitrary and different parameters are generally estimated in separate sets of experiments. In this study, an alternative method is proposed in which the electrophysiological properties of neurons are characterized by means of simplified neuron models.

Ideally, a single-neuron model should be sufficiently complex and flexible to capture, by a single change of parameters, the spiking activity of different neurons, but also simple-enough to allow robust parameter estimation [5, 6]. Detailed biophysical models with stochastic ion channel dynamics can in principle account for every aspect of single-neuron activity; however, due to their complexity, they require high computational power [5, 7–9]. While systematic fitting of detailed biophysical models is possible [10–15], most of the existing methods assume the knowledge of all the parameters that determine the dynamics of the ion channels included in the model. Overall, a reliable and efficient fitting procedure for detailed biophysical models is not known [6]. In a second class of spiking neuron models, which we call simplified threshold models, the biophysical mechanisms relevant for neural computation are not explicitly modeled, but are accounted for by phenomenological (i.e., effective) descriptions [16, 17]. Despite their simplicity, threshold models are surprisingly good at predicting single-neuron activity [6, 18–25], at least for the case of single-electrode somatic stimulation (but see [26, 27]). Nowadays, simplified threshold models are mainly used in large-scale simulations to study the emergent properties of neural circuits [28, 29]. By taking a different perspective, we will demonstrate that the same models can also serve an equally important purpose, namely to characterize the electrical properties of single neurons. In this view, simplified threshold models are interpreted as computational tools to automatically compress the information contained in a voltage recording into a set of unique and meaningful parameters. Summarizing the information of complex voltage recordings can in turn enable systematic comparisons, clustering and identification of cell types. Finally, in patch-clamp experiments aimed at studying detailed aspects of the neuronal dynamics, automated online identification of neurons could allow for *on the fly* implementation of specific stimulus sets, which are best suited for the neuron under study.

After demonstrating that a limited amount of data, and little computing time, are sufficient to fit and validate our previous Generalized Integrate-and-Fire model (GIF, see [30, 31]), we introduce an experimental protocol that, combined with automated patch-clamp technology, could make automated high-throughput single-neuron characterization possible. On the experimental side, the protocol relies on *in vitro* somatic injections of rapidly fluctuating currents that mimic natural inputs received *in vivo* at the soma of neurons. On the computational side, the protocol is based on Active Electrode Compensation [32, 33], GIF model parameter extraction [30, 31] and the spike-train similarity measure $M_{\text{d}}^{*}$ [34]. These computational methods are combined and implemented in a Python toolbox (freely available at wiki.epfl.ch/giftoolbox). The validity of our approach is finally demonstrated with two applications: i) *in silico* recordings obtained by simulating the activity of a multi-compartmental conductance-based model; and ii) *in vitro* recordings from L5 pyramidal neurons obtained using manual patch clamping. We found that fitting and validating a GIF model takes approximatively five minutes. Considering the time required to automatically establish a patch-clamp seal, the complete characterization of a single neuron can therefore be achieved in around ten minutes. We conclude that GIF models are useful not only for network simulations, but also for rapid and systematic single-neuron characterization.

## Results

The Results section is organized as follows. In the first two sections, we respectively define the GIF model and the procedures used for parameter extraction and model validation. Using artificial data generated by the GIF model itself, we then determine the amount of data and the computing time required to perform accurate parameter extraction and model validation. Based on these results, an experimental protocol is established that enables automated high-throughput characterization of single neurons. In the last sections, the validity of this protocol is verified using *in silico* recordings obtained by simulating the activity of a multi-compartmental conductance-based model [14] as well as *in vitro* recordings from layer 5 (L5) pyramidal neurons obtained using standard patch clamping. The GIF model performance is finally compared against that of a standard Generalized Linear Model (GLM) [35, 36].

### Generalized Integrate-and-Fire model

**Fig 1:**
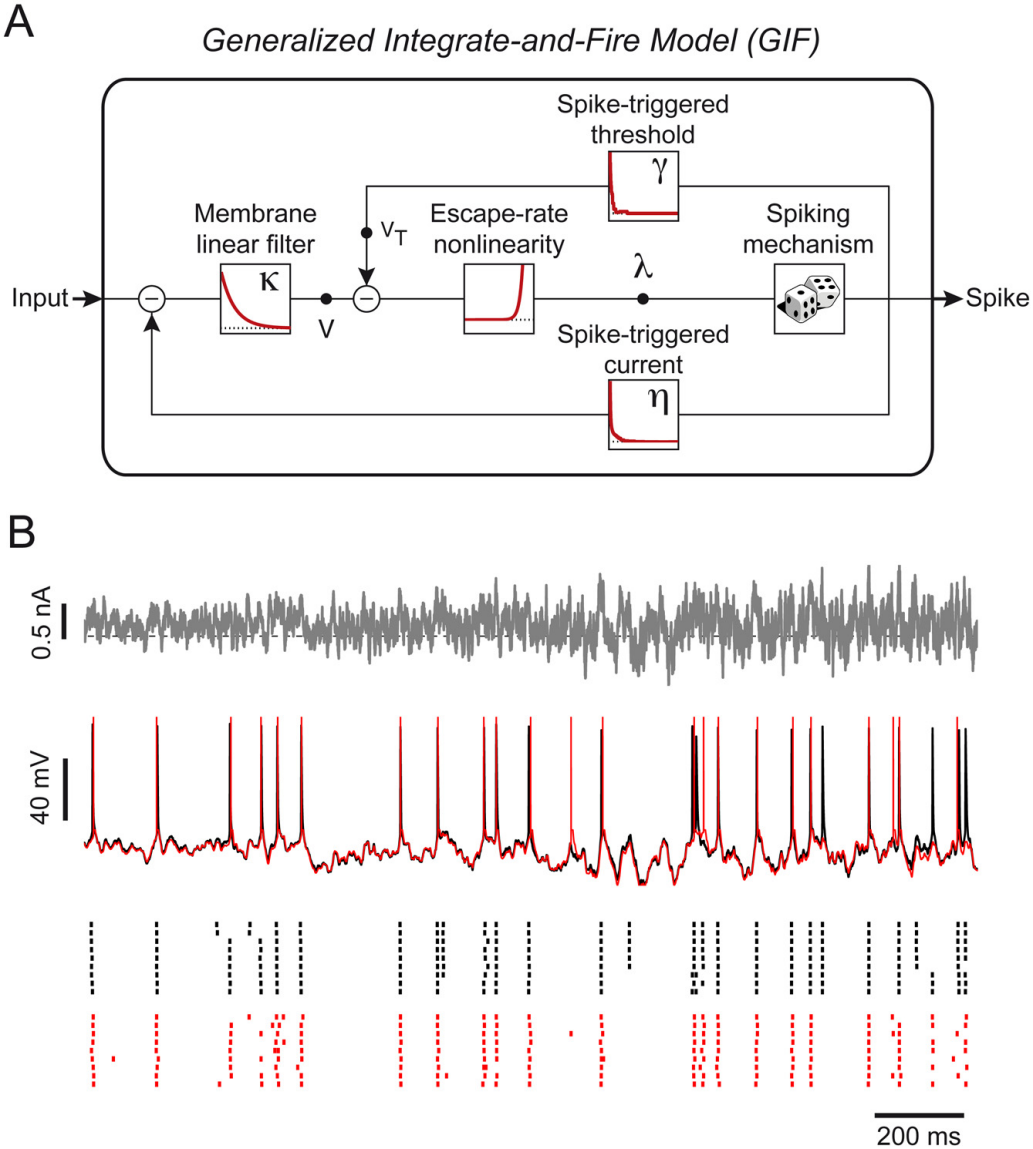
The GIF model accurately predicts both the subthreshold and the spiking activity of cortical neurons. **(A)** Block representation of the GIF model. The membrane acts as a low-pass filter *κ*(*t*) on the input current *I*(*t*) to produce the modeled potential *V*(*t*). The exponential nonlinearity (escape-rate) transforms this voltage into an instantaneous firing intensity *λ*(*t*), according to which spikes are generated. Each time a spike is emitted, both a current *η*(*t*) and a movement of the firing threshold *γ*(*t*) are triggered. **(B)** The GIF model accurately predicts the occurrence of individual spikes with millisecond precision. To evaluate the predictive power of the GIF model, the response of a L5 pyramidal neuron to a fluctuating input current (top, the horizontal dashed line represents 0 nA) has been recorded intracellularly (middle, black). The same protocol was repeated nine times to assess the reliability of the neural response (bottom, black raster). The GIF model (with parameters extracted using a different dataset) was able to accurately predict both the subthreshold (middle, red) and the spiking response (bottom, red raster) of the cell.

The GIF model discussed in this study [31, 37] is a leaky integrate-and-fire model augmented with a spike-triggered current *η*(*t*), a moving threshold *γ*(*t*) and the escape rate mechanism [38, 39] for stochastic spike emission (Fig 1A). This model is able to predict both the spiking activity and the subthreshold dynamics of individual neurons (Fig 1B), and it is flexible enough to capture the behavior of different neuronal cell types [37].

In the model, the subthreshold membrane potential *V*(*t*) evolves according to the following differential equation:

$$\begin{array}{c} C\dot{V}\left(t\right)=-g_{\text{L}}\left(V\left(t\right)-E_{\text{L}}\right)-\sum_{{\hat{t}}_{j}<t}\eta \left(t-{\hat{t}}_{j}\right)+I\left(t\right), \end{array}$$ (1)

where the parameters *C*, *g*_L_ and *E*_L_ define the passive properties of the neuron, *I*(*t*) is the input current and $\left\{{\hat{t}}_{j}\right\}$ are the spike times. According to Eq 1, the passive properties of the membrane are described by an exponential filter $\kappa (t)=\frac{R}{\tau_{\text{m}}}\text{exp}\left(\frac{-t}{\tau_{\text{m}}}\right)$, with $R=g_{\text{L}}^{-1}$ being the cell resistance and *τ*_m_ = *RC* being the membrane timescale (Fig 1A). Each time an action potential is fired, an intrinsic current with stereotypical shape *η*(*t*) is triggered. By convention, the spike-triggered current *η*(*t*) is hyperpolarizing when its amplitude is positive and depolarizing otherwise. Currents triggered by different spikes accumulate and produce spike-frequency adaptation, if *η*(*t*) > 0 (or facilitation, if *η*(*t*) < 0). The functional shape of *η*(*t*) varies among neuron types [37]. Consequently the time course of *η*(*t*) is not assumed *a priori* but is extracted from intracellular recordings. Each time a spike is emitted, the numerical integration is stopped during a short absolute refractory period *T*_ref_ and the membrane potential is reset to $V({\hat{t}}_{j}+T_{\text{ref}})=V_{\text{reset}}$.

Spikes are produced stochastically according to a point process with conditional firing intensity *λ*(*t*∣*V*, *V*_*T*_), which exponentially depends on the momentary difference between the membrane potential *V*(*t*) and the firing threshold *V*_T_(*t*) [22, 39, 40]:

$$\begin{array}{c} \lambda \left(t|V,V_{T}\right)=\lambda_{0}\cdot \text{exp}(\frac{V\left(t\right)-V_{T}\left(t\right)}{\Delta V}), \end{array}$$ (2)

where *λ*_0_ has units of *s* ^−1^, so that *λ*(*t*) is in Hz and Δ*V* defines the level of stochasticity. According to Eq 2, if Δ*V* ≠ 0, the probability of a spike to occur at a time $\hat{t}\in [t;t+\Delta t]$ is given by:

$$\begin{array}{c} P\left(\hat{t}\in \left[t;t+\Delta t\right]\right)=1-\exp(-\int_{t}^{t+\Delta t}\lambda (s)ds)\approx \lambda \left(t\right)\Delta t. \end{array}$$ (3)

In the limit Δ*V* → 0, the model becomes deterministic and action potentials are emitted at the precise moment when the membrane potential crosses the firing threshold. Importantly, the value of Δ*V* is extracted from experimental data.

Finally, the dynamics of the firing threshold *V*_*T*_(*t*) is given by:

$$\begin{array}{c} V_{T}\left(t\right)=V_{T}^{*}+\sum_{{\hat{t}}_{j}<t}\gamma \left(t-{\hat{t}}_{j}\right), \end{array}$$ (4)

where $V_{T}^{*}$ is a constant and *γ*(*t*) describes the stereotypical time course of the firing threshold after the emission of an action potential. Since the contribution of different spikes accumulates, the moving threshold defined in Eq 4 constitutes an additional source of adaptation (or facilitation). Similar to *η*(*t*), the functional shape of *γ*(*t*) is not assumed *a priori* but is extracted from intracellular recordings. All model parameters are summarized in Table 1.

**Table 1:** List of model parameters and symbols.

**GIF model**		**GLM**	
Membrane capacitance	*C*	Stimulus filter	*κ*_GLM_(*t*)
Membrane conductance	*g*_L_	Spike-history filter	*h*_GLM_(*t*)
Reversal potential	*E*_L_	Baseline activity	*E*_0_
Refractory period	*T*_ref_		
Voltage reset	*V*_reset_	**AEC**	
Threshold baseline	$V_{T}^{*}$	I-V optimal linear filter (Eq 11)	*κ*_opt_(*t*)
Threshold sharpness	Δ*V*	Electrode filter (Eq 14)	*κ*_e_(*t*)
Spike-triggered current	*η*(*t*)	Electrode time constant	*τ*_e_
Spike-triggered threshold	*γ*(*t*)		
Cell resistance	$R=g_{\text{L}}^{-1}$	**Performance evaluation**	
Membrane timescale	*τ*_m_ = *RC*	Spike-train similarity (Eq 25)	$M_{d}^{*}$
Membrane filter	*κ*(*t*)	Voltage prediction error (Eq 27)	*ϵ*_V_
Effective adaptation filter (Eq 7)	*h*(*t*)	Parameter prediction error (Eq 28)	*ϵ*_param_

### GIF model parameter extraction

**Fig 2:**
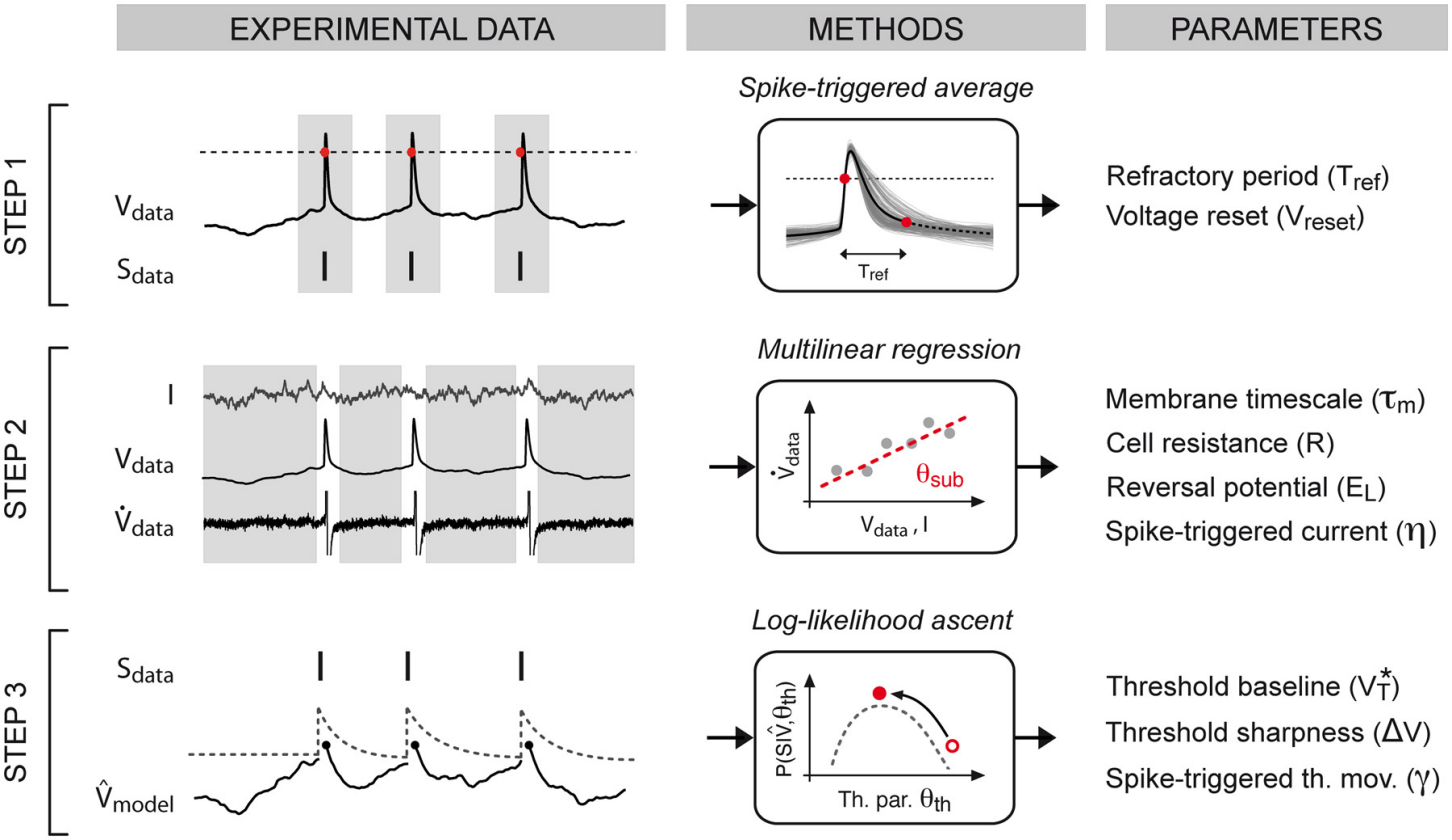
Schematic representation of the procedure used for GIF model parameter extraction. In *Step 1* (first row), the experimental spike train *S*_data_(*t*) is extracted from the voltage trace *V*_data_(*t*) using a standard threshold-crossing method (left, dashed line). Parameters related to absolute refractoriness are extracted from the average spike shape (middle). In *Step 2* (second row), given the injected current *I*_tr_(*t*) and the recorded potential *V*_data_, all the parameters *θ*_sub_ defining the dynamics of the subthreshold membrane potential (Eq 1) are extracted by performing a least-square multilinear regression on the membrane potential derivative ${\dot{V}}_{\text{data}}(t)$. Since Eq 1 does not describe the membrane potential dynamics during action potentials, all the data close to spikes are discarded. In *Step 3* (third row), the subthreshold parameters *θ*_sub_ are first used to compute the subthreshold voltage of the model ${\hat{V}}_{\text{model}}(t)$. The parameters *θ*_th_ defining the dynamics of the firing threshold (left, dashed line) are then extracted by maximizing the probability (i.e., the *log-likelihood*) that the experimental spike train *S*_data_(*t*) was produced by the model, given the subthreshold dynamics ${\hat{V}}_{\text{model}}(t)$.

Given the intracellular voltage response *V*_data_(*t*) evoked *in vitro* by a controlled input current *I*_tr_(*t*), all of the GIF model parameters are extracted from experimental data (*training set*) using a three-step procedure (Fig 2) that we previously introduced [30, 31]. A detailed description of the fitting procedure can be found in the Materials and Methods section.

In *Step 1* (Fig 2, Step 1), the experimental spike train $S_{\text{data}}=\{{\hat{t}}_{j}\}$ is first defined as the collection of instants ${\hat{t}}_{j}$ at which *V*_data_(*t*) crossed a certain threshold from below. The average spike shape *V*_STA_(*t*) is then obtained by computing the spike-triggered average (STA) of *V*_data_(*t*). Depending on the cell type (i.e., depending on the average spike shape), the absolute refractory period *T*_ref_ is fixed to a certain value and the reset potential is computed as *V*_reset_ = *V*_STA_(*T*_ref_). In the GIF model, a period of absolute refractoriness can alternatively be implemented by setting the first milliseconds of the spike-triggered threshold movement *γ*(*t*) to very large values. For this reason, as long as *T*_ref_ remains smaller than the shortest interspike interval (ISI) observed in the data, its precise value is not critical. A sensible choice is to set *T*_ref_ about twice the spike width at half maximum.

In *Step 2* (Fig 2, Step 2), the first-order temporal derivative of the experimental voltage ${\dot{V}}_{\text{data}}(t)$ is estimated by finite differences and the parameters *θ*_sub_ = {*C*, *g*_L_, *E*_L_, *η*(*t*)} determining the membrane potential dynamics are extracted by fitting Eq 1 on ${\dot{V}}_{\text{data}}(t)$. This is done by exploiting the knowledge of the experimental voltage *V*_data_(*t*) and the external input *I*_tr_(*t*). To avoid *a priori* assumptions on the functional shape of the spike-triggered current, *η*(*t*) is expanded in a linear combination of rectangular basis functions. Consequently, optimal parameters minimizing the sum of squared errors between $\dot{V}(t)$ and ${\dot{V}}_{\text{data}}(t)$ can be efficiently obtained by solving a multilinear regression problem [22] (cf. Eqs 17–18).

In *Step 3* (Fig 2, Step 3), the parameters estimated so far are first used to compute the subthreshold membrane potential of the model ${\hat{V}}_{\text{model}}(t)$. For that, Eq 1 is numerically solved by enforcing adaptation currents *η*(*t*) at all the observed spike times $\left\{{\hat{t}}_{j}\right\}$. Given ${\hat{V}}_{\text{model}}(t)$, the parameters $\theta_{\text{th}}=\{V_{T}^{*},\Delta V,\gamma (t)\}$ defining the firing threshold dynamics (cf. Eqs 2–4) are then extracted by maximizing the probability (i.e., the *log-likelihood*) of the experimental spike train *S*_data_(*t*) being produced by the GIF model (cf. Eqs 20–21). Similar to *η*(*t*), the spike-triggered threshold movement is extracted by expanding *γ*(*t*) in a linear combination of rectangular basis functions. Since the parameters *λ*_0_ and $V_{T}^{*}$ are redundant, *λ*_0_ is fixed to 1 Hz. With the exponential function in Eq 2, the *log-likelihood* to maximize is guaranteed to be a concave function of *θ*_th_[41] and the optimization problem can be solved using standard gradient ascent techniques. The method used in this last step closely resembles the standard GLM fitting procedure [35, 36]. However, here, by exploiting the information contained in the subthreshold dynamics of the membrane potential, the maximum likelihood approach is specifically used to infer the dynamics of the firing threshold. In contrast to GLMs, the GIF model can consequently disentangle adaptation processes mediated by intrinsic currents and threshold movements.

### GIF model validation

To obtain a high-throughput pipeline for GIF model parameter extraction, the method described in the previous section has to be complemented with a validation protocol designed to automatically detect and discard trials in which the fitting procedure fails. Good spiking neuron models should be able to accurately predict the occurrence of individual action potentials with millisecond precision [6]. To take into account the stochastic nature of single neurons [42], we designed a validation protocol based on the measurement of the model performance in predicting spike emission probability. After the acquisition of the *training dataset* used for parameter extraction, a new set of recordings (*test dataset*) is performed in which single neurons are stimulated repetitively with a test current *I*_test_(*t*). The resulting set of experimental spike trains is then compared against a set of spike trains predicted by repetitive simulations of the GIF model. To obtain a quantitative measure of the model’s predictive power, the similarity $M_{d}^{*}$ [34] between the two sets of spike trains is computed (Materials and Methods). $M_{d}^{*}$ takes values between 0 and 1, where $M_{d}^{*}=0$ indicates that the model is unable to predict any of the experimental spikes and $M_{d}^{*}=1$ indicates a perfect match. Importantly, $M_{d}^{*}$ avoids the *small-sample bias* known to occur when measuring the similarity between small groups of spike trains as well as the *deterministic bias* known to favor noise-free models [34].

### Testing GIF model parameter extraction and validation on artificial data

**Fig 3:**
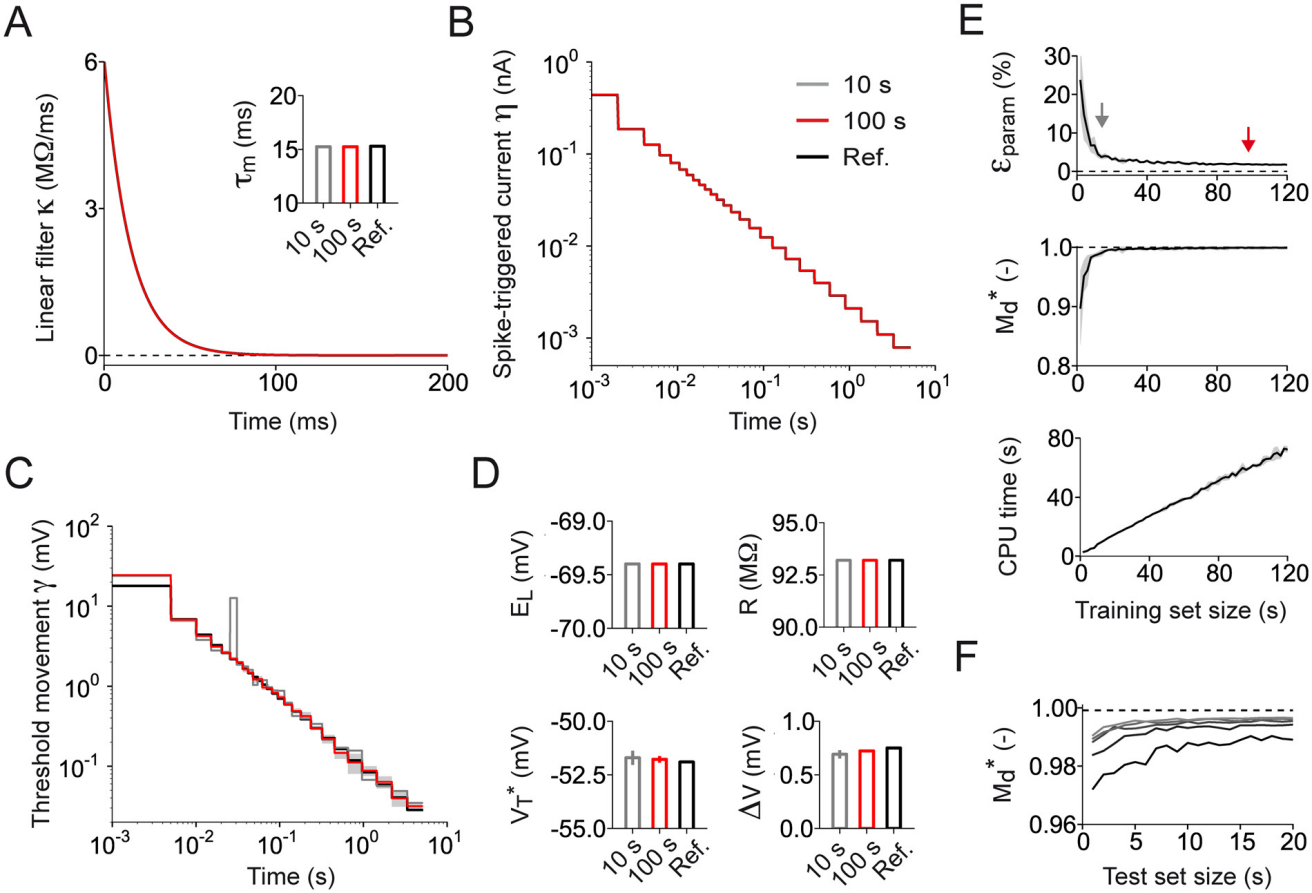
Validation of the procedure used for GIF model parameter extraction. **(A)-(D)** GIF model parameters used to generate the artificial data (black) recovered using a training set of *T*_tr_ = 10 s (gray) and *T*_tr_ = 100 s (red). Error bars and shaded areas represent one standard deviation obtained using five different data sets. In case of perfect agreement, black lines, gray lines and shaded areas are not visible. **(A)** Membrane filter *κ*(*t*). Inset: membrane timescale *τ*_m_ = *C*/*g*_L_. **(B)** Spike-triggered current *η*(*t*). **(C)** Spike-triggered movement of the firing threshold *γ*(*t*). **(D)** Reversal potential (*E*_L_, top left); cell resistance ($R=g_{\text{L}}^{-1}$, top right); threshold baseline ($V_{\text{T}}^{*}$, bottom left) and threshold sharpness (Δ*V*, bottom right). **(E)** Estimation error *ϵ*_param_ on model parameters (upper panel), performance on spike-timing prediction $M_{d}^{*}$ (middle panel) and computing time required for parameter extraction (lower panel) as a function of the training set size *T*_tr_. Gray areas indicate one standard deviation across different artificial datasets generated using the same reference parameters. Gray and red arrows indicate the performance obtained with a training set of 10 s and 100 s, respectively. **(F)** Reliability of the validation procedure as a function of the number of repetitions *n*_test_ and the duration *T*_test_ of the test current. For different values of *n*_test_ and *T*_test_, $M_{d}^{*}$ was computed 1000 times using different test currents. Consistent with the result that $M_{d}^{*}$ corrects the small-sample bias, the mean value of $M_{d}^{*}=0.998$ (dashed line) obtained across repetitions of different test currents did not depend on *n*_test_ and *T*_test_. The continuous lines represent the 0.25-quantiles of the $M_{d}^{*}$ distribution obtained with *n*_test_ = {3,6,9,12,15} (from dark to light gray) and indicate that the reliability of the measure increases with *n*_test_ and *T*_test_.

To estimate the amount of data required to perform GIF model parameter extraction, we first tested our fitting procedure on an artificial training set generated by simulating the response of a GIF model to a fluctuating current *I*(*t*). The choice of reference parameters (Fig 3A–3D, black) was based on previous results [31]. In particular, both the spike-triggered current *η*(*t*) and the threshold movement *γ*(*t*) were defined as a linear combination of *K* = 26 log-spaced rectangular basis functions approximating a power-law decay over 5 seconds [31, 43]. Overall, the reference model had 59 parameters: 31 were related to the subthreshold dynamics and 28 to the firing threshold.

The input current *I*(*t*) used to build the artificial training set was generated at Δ*T* ^−1^ = 20 kHz by numerically solving the stochastic differential equation $\tau \dot{I}=-I+I_{0}+\sqrt{2\tau }\sigma (t)\xi (t)$ in discrete time

$$\begin{array}{c} I\left(t+\Delta T\right)=I\left(t\right)+\frac{I_{0}-I\left(t\right)}{\tau }\cdot \Delta T+\sqrt{\frac{2\sigma^{2}\Delta T}{\tau }}\cdot 𝓝\left(0,1\right), \end{array}$$ (5)

where *ξ*(*t*) is a Gaussian white-noise process generated by independently sampling from a Normal distribution 𝓝(0,1), *τ* = 3 ms is the characteristic timescale on which the input fluctuates, *I*_0_ defines the mean input and *σ*(*t*) is the time-dependent standard deviation of *I*(*t*). Ornstein-Uhlenbeck processes (i.e. stationary filtered Gaussian processes) have been extensively used to model the input current received *in vivo* at the soma of neocortical neurons [44]. Here, we relaxed the assumption of stationarity by modulating the variance of the input with a periodic oscillation [43] given by:

$$\begin{array}{c} \sigma \left(t\right)=\sigma_{0}\left(1+\Delta \sigma \sin\left(2\pi ft\right)\right), \end{array}$$ (6)

where *σ*_0_ and Δ*σ* are constants and *f* = 0.2 Hz is the modulation frequency. An input current with non-stationary statistics drives the neurons through different regimes producing broad ISI distributions that better constrain the fit of adaptation processes. The input parameters *I*_0_, *σ*_0_ and Δ*σ* were adjusted to generate an artificial training set in which the GIF model emitted spikes at an average firing rate of 10 Hz oscillating over 5 seconds between around 7 and 13 Hz.

The fitting procedure illustrated in Fig 2 was then applied to recover the reference parameters of the GIF model used to generate the artificial dataset (Fig 3A–3D, black). To estimate the amount of data required to guarantee a high degree of accuracy, this operation was repeated several times by varying the size of the training set *T*_tr_ (i.e., the duration of the input current *I*(*t*)). Fig 3A–3D shows a comparison between the reference parameters and the results obtained by fitting a training set of *T*_tr_ = 10 seconds (gray) and *T*_tr_ = 100 seconds (red). Overall, we found that 100 seconds were sufficient to accurately recover the reference parameters. To quantify the accuracy of the fit, we computed the mean error *ϵ*_param_ on model parameters as a function of *T*_tr_ (see Materials and Methods). The results indicate that the minimum amount of data required for accurate parameter extraction is 30–40 seconds. In particular, we found that 100 seconds were sufficient to limit the error to *ϵ*_param_ < 2.0% (Fig 3E, top). The great accuracy with which the fitted model was able to predict the spiking activity of the reference model ($M_{d}^{*}=0.998$) confirmed the goodness of this fit (Fig 3E, middle). To achieve high-throughput and perform parameter extraction *on the fly*, it is crucial to minimize the computing time (CPU time) required for the fit. We measured the CPU time as a function of the training set duration *T*_tr_ (Fig 3E, bottom) and we found that accurate parameter extraction from a training set of *T*_tr_ = 100 seconds requires around 60 seconds of computing. We concluded that GIF model parameter extraction is suitable for high-throughput.

A second time-consuming procedure that has to be analyzed is the validation protocol. To quantify the predictive power of the fitted model, the reference model was stimulated with repetitive injections of a test current *I*_test_(*t*) generated according to Eqs 5–6. To estimate the number of repetitions *n*_test_ and the duration *T*_test_ of the test current required to obtain a reliable estimate of the model predictive power, the similarity measure $M_{d}^{*}$ was computed multiple times using different values of *n*_test_ and *T*_test_ (Fig 3F). On average, the value of $M_{d}^{*}$ was independent of both the input current duration and the number of repetitions, confirming that the spike-train metrics $M_{d}^{*}$ successfully eliminates the *small sample bias* [34]. We measured the variability of $M_{d}^{*}$ across validation procedures performed with different realizations of *I*_test_(*t*) and found that the reliability of $M_{d}^{*}$ increased with both the number of repetitions *n*_test_ and the duration of the test current *T*_test_ (Fig 3F). Spike-triggered processes can last for several seconds [31, 43]. This sets a constraint on the minimal duration of both the test current *I*_test_(*t*) and the interstimulus interval. By taking into account these constraints, we concluded that, while respecting high-throughput constraints, a validation protocol based on nine injections of a 10-second current guarantees a reliable estimation of the model’s predictive power (Fig 3F).

### A protocol for automated high-throughput single-neuron characterization

**Fig 4:**
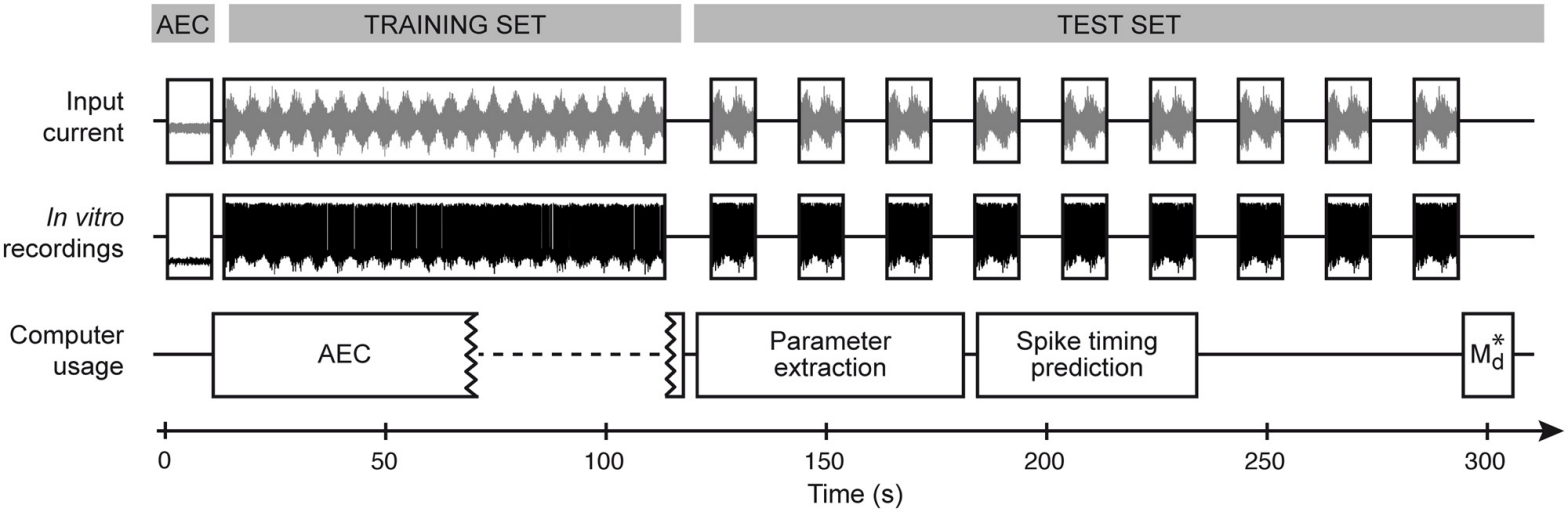
Schematic representation of the protocol for high-throughput single-neuron characterization. To characterize the properties of the electrode required for AEC, the experimental protocol starts with the injection of a short subthreshold current. While the filtering properties of the patch clamp are estimated (AEC box, left), the training dataset is collected. After training set collection, the raw data are preprocessed with AEC (AEC box, right). Then, in parallel with GIF model parameter extraction and successive spike timing prediction, the test dataset is collected by injecting nine repetitions of the same time-dependent current. Finally, after complete acquisition of the test set, the similarity measure $M_{\text{d}}^{*}$ between the observed and the predicted spike trains is computed. Overall, GIF model parameter extraction and validation requires around five minutes.

Based on the results reported in the previous section, we designed a protocol for the fit and the validation of GIF models on *in vitro* intracellular recordings (Fig 4). The protocol is conceptually divided in two phases. In the first part, a *training set* is acquired by recording the single-neuron response to a fluctuating input *I*_tr_(*t*) lasting for *T*_tr_ = 100 seconds and generated according to Eqs 5–6. These data are then used for parameter extraction. In the second part of the protocol, nine repetitive injections of a new 10-second current *I*_test_(*t*) are performed with an interstimulus interval of 10 seconds, so as to allow the cell to recover. These data (*test set*) are then used to quantify the predictive power of the GIF model with the spike-train similarity measure $M_{d}^{*}$. Since all the computations required for parameter extraction and model validation can be performed *on the fly*, the whole protocol requires 5 minutes and is suitable for high-throughput.

Current-clamp experiments in which the same electrode is used both for stimulating and recording from single neurons are biased due to the voltage drop across the electrode [32]. To remove this bias, intracellular recordings are preprocessed using a technique called Active Electrode Compensation (AEC, refs. [32, 33], see Materials and Methods). To perform AEC, the filtering properties of the electrode have to be estimated. For that, an additional 10-second subthreshold current injection is performed before the acquisition of the *training set* (Fig 4).

### Testing the high-throughput protocol on *in silico* recordings

A different class of models used to describe the electrical activity of individual neurons includes the so called multi-compartment conductance-based models (or detailed biophysical models). In contrast to point-neuron models, detailed biophysical models account for the intricate morphology of both dendritic and axonal arborizations and explicitly describe the dynamics of a large variety of ion channels mediating active currents. Both aspects are likely to play a role in single-neuron information processing [45, 46]. A detailed biophysical model (DBM) has recently been proposed that captures several features of L5b thick-tufted pyramidal neurons [14]. In particular, this model includes active dendrites and describes the interactions between Na^+^-spiking at the soma, back-propagating action potentials and Ca^2+^-spikes generated at the distal apical dendrites.

**Fig 5:**
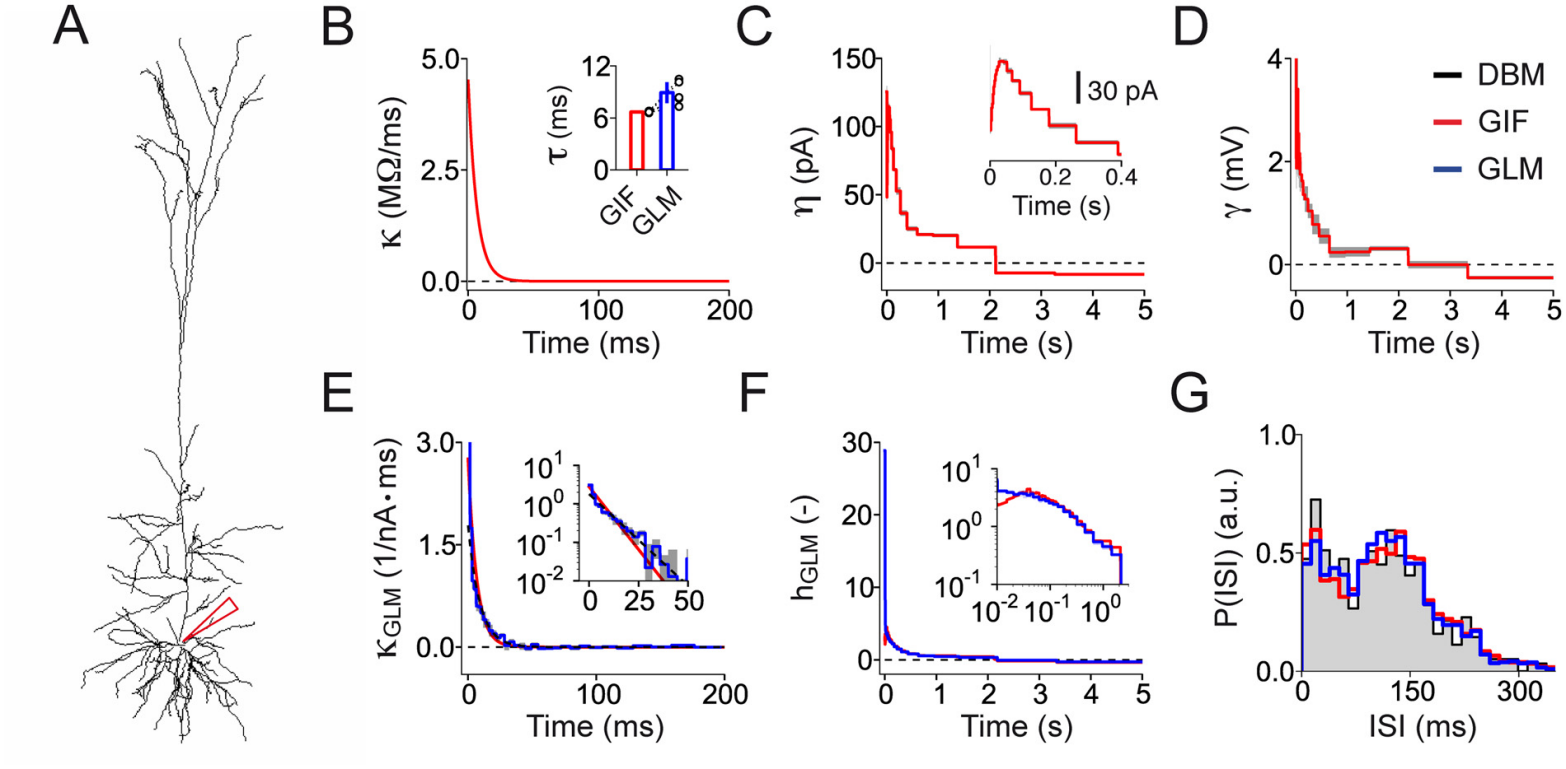
Testing GIF model parameter extraction on *in silico* recordings from a detailed biophysical model: optimal GIF model parameters. **(A)** Reconstructed morphology of the detailed biophysical model (DBM, ref. [14]) used to validate the protocol for high-throughput single-neuron characterization. The recording site is indicated by the red pipette. **(B)-(D)** GIF model parameters extracted from *in silico* recordings obtained by simulating the DBM response to a somatic current injection. The filters obtained by averaging the parameters extracted from five independent training sets of *T*_tr_ = 100 s each are shown in red. Gray areas indicate one standard deviation. **(B)** Membrane filter *κ*(*t*). Inset: comparison between the membrane timescale extracted using a GIF model and the timescale of the GLM linear filter (c.f., exponential fit of *κ*_GLM_(*t*) in panel *E*). Each couple of open circles indicates the timescale extracted from a specific training set. Bar plots represent the mean and one standard deviation across training sets (*τ*_m_ = 6.7 ms, s.d. 0.1 ms, GIF; *τ*_m_ = 8.9 ms, s.d. 1.3 ms, GLM). **(C)** Spike-triggered current *η*(*t*). Inset: zoom on the first 400 ms. **(D)** Spike-triggered movement of the firing threshold *γ*(*t*). **(E)-(F)** GLM parameters extracted from the same *in silico* recordings used to fit the GIF model. Average filters are shown in blue. Gray areas indicate one standard deviation across training sets. **(E)** Linear filter *κ*_GLM_(*t*) (blue) and exponential fit (dashed black). For comparison, a rescaled version of the membrane filter *κ*(*t*) is shown in red. Inset: same data displayed on semi-logarithmic scales. **(F)** Spike-history filter *h*_GLM_(*t*). For comparison, a rescaled version of the GIF model effective filter *h*(*t*) (Eq 7) is shown in red. Inset: same data displayed on double-logarithmic scales. **(G)** ISI distributions computed using the *test set* data (black line and gray area) the GIF model prediction (red) and the GLM prediction (blue).

To validate our procedure for high-throughput single-neuron characterization, the protocol described in Fig 4 was tested *in silico* by simulating the DBM response to a set of current injections (Fig 5A, see Materials and Methods). The input parameters were calibrated to obtain an average firing rate of 10 Hz with slow rate fluctuations between 7 and 13 Hz. Moreover, to model stochastic spike emission, a source of noise was introduced by corrupting the input current with some additive white-noise (see Materials and Methods). Capturing the DBM spiking response to dendritic injections goes beyond the scope of this study. Since we are ultimately interested in automatic somatic patching, all *in silico* experiments were preformed by delivering the current at the somatic compartment (Fig 5A). DBM somatic recordings were then used to perform GIF model parameter extraction (Fig 5B–5D). Compared with previous results from *in vitro* recordings in L5 pyramidal neurons [30, 31], the membrane filter *κ*(*t*) was characterized by a relatively short timescale (*τ*_m_ = 6.7 ms, s.d. 0.1 ms, Fig 5B). GIF model parameter extraction also revealed the presence of a long-lasting adaptation current (Fig 5C) as well as a long-lasting spike-triggered movement of the firing threshold (Fig 5D). Consistent with the tendency of L5b pyramidal neurons to produce bursts of action potentials (ref. [14] and Fig 5G), the activation of the spike-triggered current was not instantaneous.

According to cable theory [47], the large number of dendritic branches explicitly modeled in the DBM, is expected to manifest itself in a membrane filter *κ*(*t*) decaying over multiple timescales. To verify the accuracy of the single-exponential assumption and to compare the GIF model performance against a reference model, we also used the *in silico* recordings to fit a GLM [35, 36], (Fig 5E and 5F). In the GLM, the linear filter *κ*_GLM_(*t*) acting on the input current is not assumed *a priori* to be an exponential function and its shape is extracted from experimental data using a non-parametric method (see Materials and Methods). We found that the GLM filter *κ*_GLM_(*s*) and the membrane filer *κ*(*t*) of the GIF model were in good agreement (Fig 5E), suggesting that complex dendritic morphologies weakly affect temporal integration at the somatic compartment. Further quantitative evidence was provided by fitting *κ*_GLM_(*t*) with a single exponential function and comparing the resulting timescale against *τ*_m_ (Fig 5B, inset). The GLM spike-history filter *h*_GLM_(*t*) extracted from *in silico* recordings (Fig 5F) was also in good agreement with the effective adaptation filter *h*(*t*) of the GIF model [31, 37]:

$$\begin{array}{c} h\left(t\right)=\int_{0}^{\infty }\kappa \left(s\right)\eta \left(t-s\right)ds+\gamma \left(t\right). \end{array}$$ (7)

This result confirmed that *h*_GLM_(*t*) combines, but cannot disentangle, the effects of the adaptation current *η*(*t*) and the movement of the firing threshold *γ*(*t*). In contrast to GIF models, GLMs do not model absolute refractoriness with a dead time followed by a voltage reset. This explains why, during the first milliseconds, *h*_GLM_(*t*) is much larger than *h*(*t*) (Fig 5F). Finally, consistent with previous results that in L5 pyramidal neurons spike-frequency adaptation occurs on multiple timescales [31, 43], we noticed that both *h*(*t*) and *h*_GLM_(*t*) were approximatively linear on double logarithmic scales (Fig 5F, inset).

**Fig 6:**
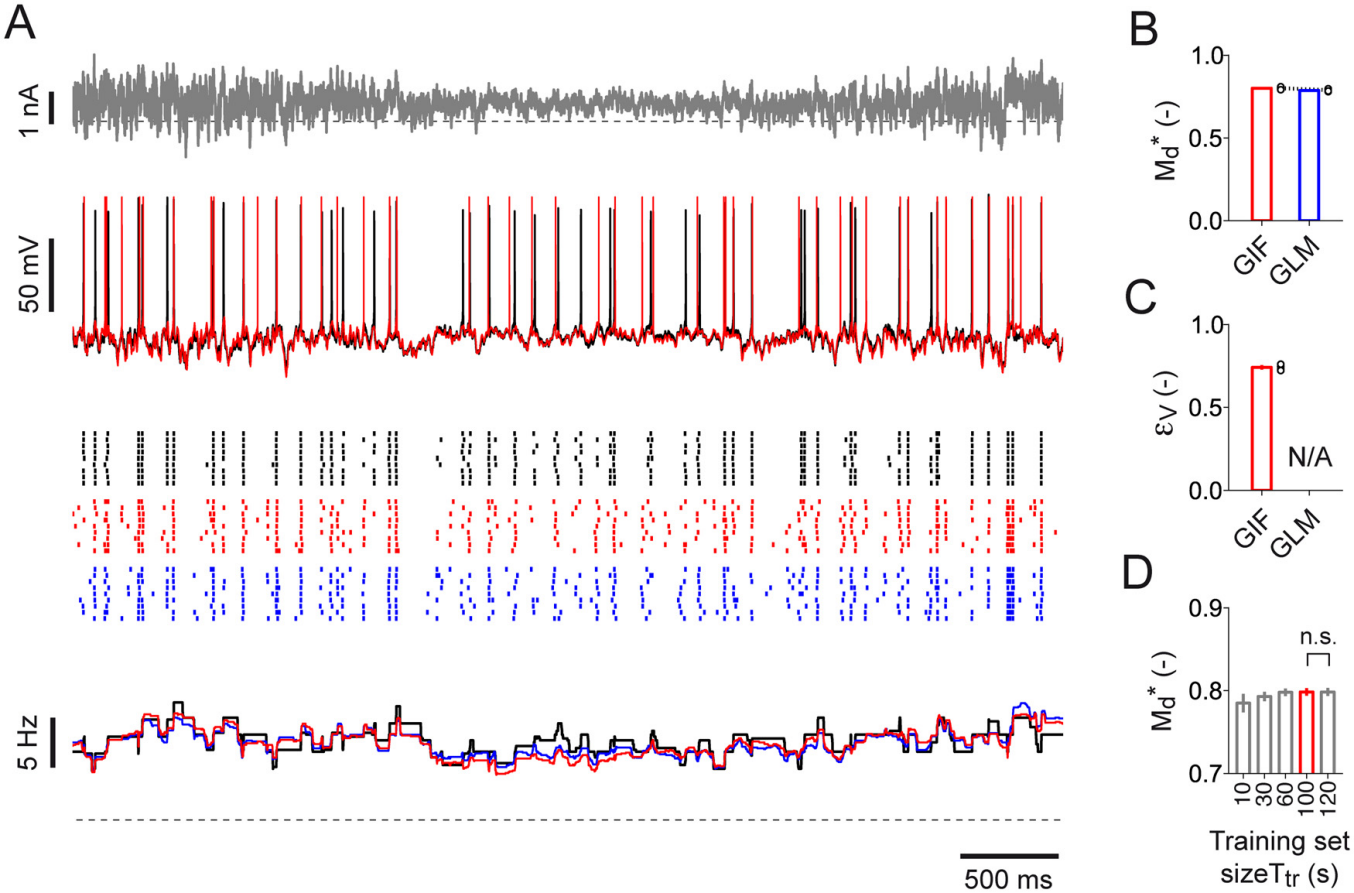
Testing GIF model parameter extraction on *in silico* recordings from a detailed biophysical model: GIF model validation. **(A)** Fraction of the input current *I*_test_(*t*) (top, gray) used for model validation; typical DBM response evoked by a single current injection (middle, black); DBM spiking activity in response to nine repetitive injections of the same input (bottom, black raster); PSTH constructed by averaging the nine spike trains smoothed with a rectangular window of 500 ms (bottom, black line). GIF model and GLM predictions are shown in red and blue, respectively. Dashed black lines represent 0 nA (top) and 0 Hz (bottom). **(B)-(D)** Performance comparison between GIF model (red) and GLM (blue) in predicting the DBM activity. Parameter extraction and model validation were repeated five times using different datasets. Each couple of open circles indicates the performance obtained by both models on a specific dataset. Bar plots indicate the mean and one standard deviation across repetitions. **(B)** Spike-timing prediction as quantified by $M_{d}^{*}$ with precision Δ = 4 ms. **(C)** Mean prediction error *ϵ*_V_ on subthreshold membrane potential fluctuations. The GLM does not explicitly model the subthreshold membrane potential dynamics and is therefore not applicable (N/A). **(D)** GIF model spike-timing prediction ($M_{d}^{*}$, with precision Δ = 4 ms) as a function of the training set size used for parameter extraction. Increasing the duration of the training set from 100 s to 120 s does not improve the GIF model predictive power ($M_{d}^{*}$ = 0.80, s.d. 0.01, *T*_tr_ = 100 s; $M_{d}^{*}$ = 0.80, s.d. 0.01, *T*_tr_ = 120 s; *n* = 10, paired Student *t*-test, *t*_4_ = 0.05, *p* = 0.97; n.s. > 0.05).

The predictive power of both the GIF model and the GLM was then assessed on a *test set* obtained by simulating the DBM response to nine repetitive injections of a new 10-second current (Fig 6A). Both models achieved a similar performance and were able to predict around 80% of the spikes emitted by the DBM (temporal precision Δ = 4 ms; $M_{d}^{*}$ = 0.80, s.d. 0.01, GIF; $M_{d}^{*}$ = 0.79, s.d. 0.01, GLM; Fig 6B). Compared to the GLM, the GIF model presented two advantages. First, the GIF model, but not the GLM, explicitly modeled the dynamics of the membrane potential and could therefore predict the DBM subthreshold voltage with an average root mean squared error (RMSE) of 3.4 mV, s.d. 0.03 mV (variance explained *ϵ*_V_ = 74.3 %, s.d. 1.1%; Fig 6C). Second, the time required to perform parameter extraction was faster for the GIF model than for the GLM (*T*_CPU_ = 86 s, GIF; *T*_CPU_ = 143 s, GLM).

Repeating the entire protocol by varying the duration of *I*_tr_(*t*) confirmed that a training set of *T*_tr_ = 100 s was sufficient to ensure convergence of the fitting procedure (Fig 6D). Overall, these results suggest that, despite their simplicity, modern point-neuron models are capable of predicting most of the spikes emitted by a detailed biophysical model in response to complex somatic current injections.

### Testing the high-throughput protocol on *in vitro* patch-clamp recordings

To confirm the results reported in the previous section, the protocol for high-throughput single-neuron characterization was further tested using standard current-clamp *in vitro* recordings from L5 pyramidal neurons (see Materials and Methods). At the beginning of the experiment, the input current was calibrated to obtain an average firing rate of 10 Hz with amplitude fluctuations between 7 and 13 Hz. For that, we set Δ*σ* = 0.5, *I*_0_ = *σ*_0_ and adjusted *I*_0_ in order to obtain an average firing rate of around 10 Hz. While this simple approach works well for L5 pyramidal neurons, different cell types might require a more involved calibration protocol in which *I*_0_ ≠ *σ*_0_. In these cases, an alternative solution consist of: i) temporarily setting *I*_0_ = 0 pA and looking for two values of *σ*_0_, denoted $\sigma_{0}^{\text{min}}$ and $\sigma_{0}^{\text{max}}$, giving rise to subthreshold voltage fluctuations *σ*_V_ of desired magnitudes (e.g., $\sigma_{\text{V}}^{\text{min}}\approx 3$ mV and $\sigma_{\text{V}}^{\text{max}}\approx 7$ mV); ii) set $\sigma_{0}=(\sigma_{0}^{\text{min}}+\sigma_{0}^{\text{max}})/2$ and $\Delta \sigma =(\sigma_{0}^{\text{max}}-\sigma_{0}^{\text{min}})/2\sigma_{0}$; iii) adjust *I*_0_ to obtain an average firing rate of around 10 Hz.

**Fig 7:**
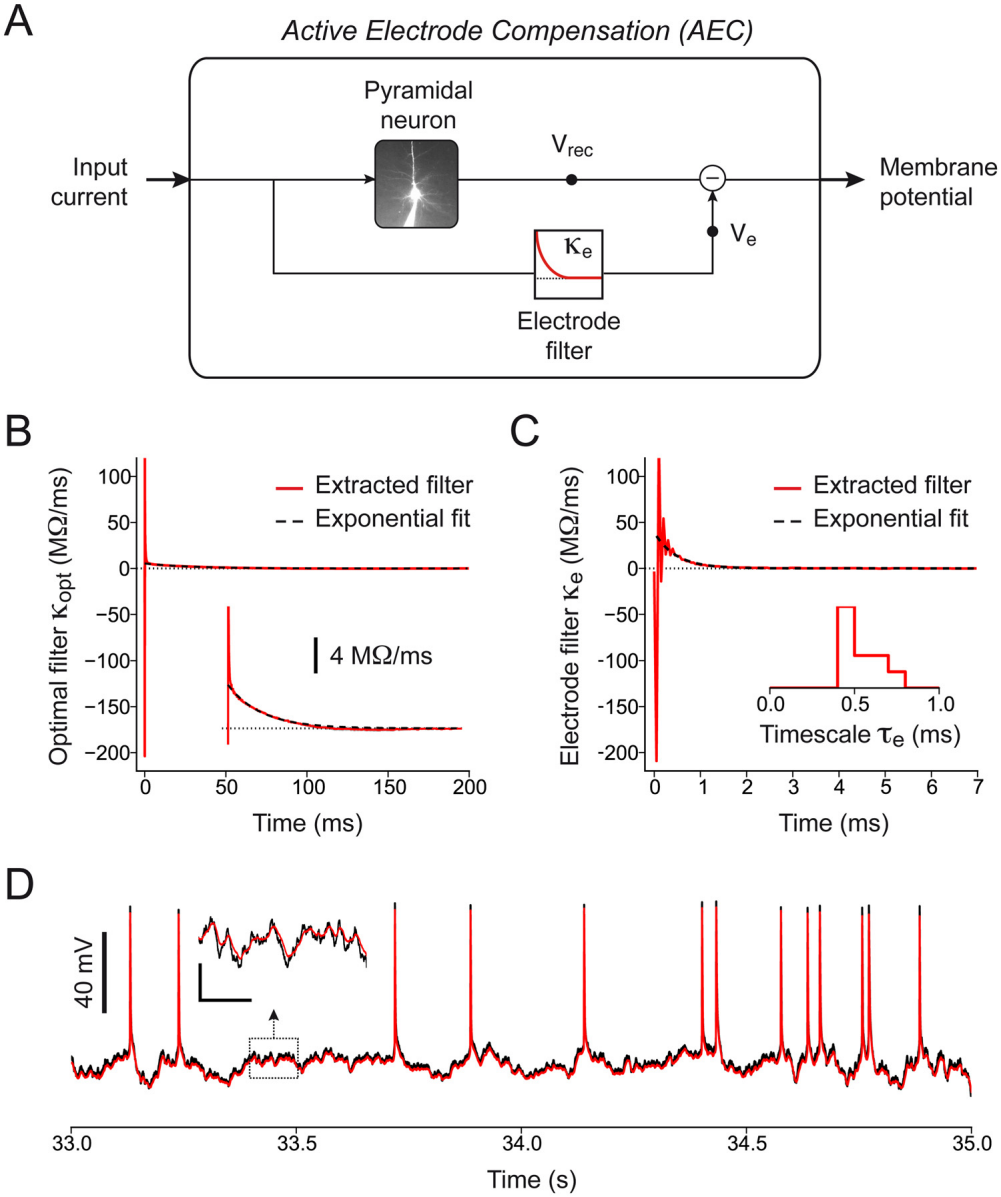
Data preprocessing: Active Electrode Compensation. **(A)** Schematic representation of the Active Electrode Compensation technique used to correct for the experimental bias known to occur when the same patch-clamp electrode is used to simultaneously inject and record from a single neuron. The artifactual voltage *V*_e_(*t*) across the pipette is estimated by filtering the input current *I*(*t*) with the electrode filter *κ*_e_(*t*). The intracellular membrane potential *V*_data_(*t*) if finally obtained by subtracting the artifactual voltage *V*_e_(*t*) from the recorded signal *V*_rec_(*t*). **(B)** Typical optimal linear filter *κ*_opt_(*t*) between the subthreshold input current *I*_sub_(*t*) and the recorded signal *V*_sub_(*t*). To estimate the electrode filter, an exponential fit is performed on the tail of *κ*_opt_(*t*) (dashed black). Inset: Magnification of the y-axis illustrating the good accuracy of the exponential fit (dashed black) on the tail of the optimal linear filter *κ*_opt_(*t*) (red). **(C)** Typical electrode filter *κ*_e_(*t*) obtained by subtracting the exponential fit from the optimal linear filter *κ*_opt_(*t*) (see panel *B*). Since *in vitro* recordings were performed with the standard bridge compensation technique, the electrode filter *κ*_e_(*t*) is characterized by a strong initial negative peak. The characteristic timescale of the electrode filter *τ*_e_ was measured by performing an exponential fit (dashed black) on *κ*_e_(*t*). Inset: distribution of the electrode timescales *τ*_e_ measured in ten different recordings included in this study. **(D)** Comparison between recorded signal *V*_rec_(*t*) (black) and membrane potential *V*_data_(*t*) (red) obtained after AEC. Inset: zoom indicating that AEC operates as a low-pass filter by removing high-frequency components from the acquired signal. Scale bars: 30 ms, 5 mV.

Since the same patch-clamp electrode was used to simultaneously stimulate and record from single neurons, the acquired signal *V*_rec_(*t*) is a biased version of the real membrane potential *V*_data_(*t*) [32, 33]. This bias is due to the voltage drop *V*_e_(*t*) across the patch-clamp electrode and was removed using a technique called Active Electrode Compensation (AEC, see Materials and Methods and Fig 7A). In AEC [32, 33], the electrode is modeled as an arbitrarily complex linear filter *κ*_e_(*t*) estimated at the beginning of the experiment from the optimal linear filter *κ*_opt_(*t*) between a 10-second subthreshold current *I*_sub_(*t*) and the recorded response *V*_sub_(*t*) (Fig 7B). For all subsequent injections, we estimated the voltage drop across the electrode *V*_e_(*t*) by convolving the input current with the electrode filter *κ*_e_(*t*) (Fig 7C). We finally recovered the membrane potential *V*_data_(*t*) by subtracting *V*_e_(*t*) from the recorded signal *V*_rec_(*t*) (Fig 7A and 7D):

$$\begin{array}{c} V_{\text{data}}\left(t\right)=V_{\text{rec}}\left(t\right)-V_{\text{e}}\left(t\right). \end{array}$$ (8)

According to our high-throughput protocol, the training set was compensated only after its complete acquisition. With this strategy, the time-consuming procedure required to estimate the electrode filter can be performed during the acquisition of the training set (see Fig 4), limiting the total duration of the protocol. Consistent with previous results [31], we found that the electrode filter *κ*_e_(*t*) decayed on a very rapid timescale *τ*_e_ = 0.54 ms, s.d. 0.11 ms (Fig 7C). Consequently, AEC acted on the raw data as a low-pass filter with a cutoff frequency of around 2 kHz.

**Fig 8:**
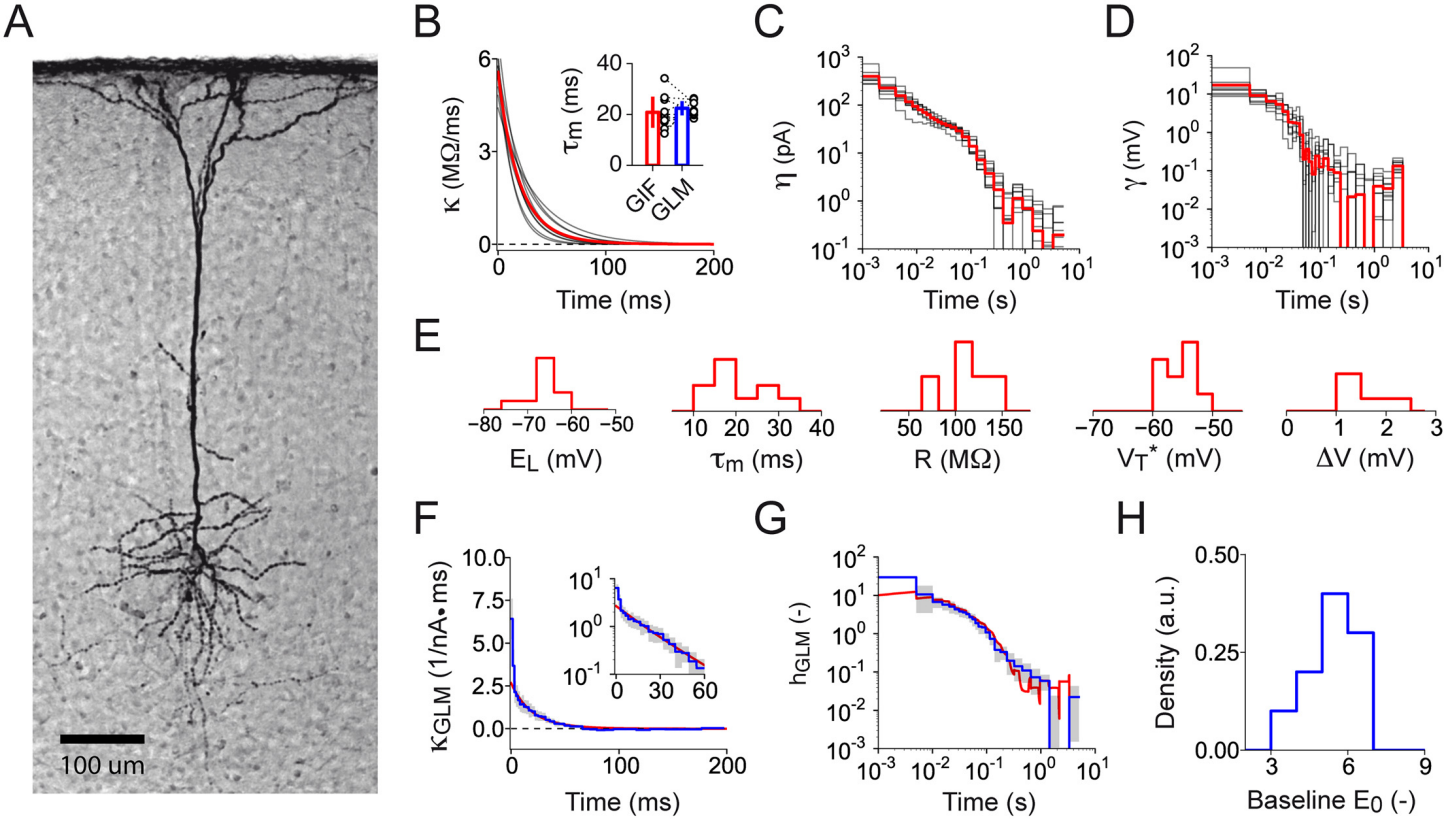
Testing the protocol for high-throughput single-neuron characterization on *in vitro* patch-clamp recordings: optimal GIF model parameters. **(A)** Staining of a biocytin-filled L5 pyramidal neuron included in this study. **(B)-(E)** GIF model parameters extracted from ten L5 pyramidal neurons. Average filters are shown in red. Gray lines show the results from individual neurons. **(B)** Membrane filter *κ*(*t*). Inset: comparison between the characteristic timescale of *κ*(*t*) and the slow timescale *τ*_slow_ of *κ*_GLM_(*t*) (see panel *F*). Each couple of open circles indicates the parameters measured in a single neuron. Bar plots indicate the mean and one standard deviation across neurons. **(C)** Spike-triggered current *η*(*t*) displayed on double-logarithmic scales. **(D)** Spike-triggered movement of the firing threshold *γ*(*t*) displayed on double-logarithmic scales. **(E)** Histograms of GIF model parameters extracted from ten L5 pyramidal neurons. From left to right: reversal potential, *E*_L_; membrane timescale, *τ*_*m*_ = *C*/*g*_L_; cell resistance, $R=g_{\text{L}}^{-1}$; firing threshold baseline, $V_{\text{T}}^{*}$; firing threshold sharpness, Δ*V*. **(F)-(H)** GLM parameters extracted from ten L5 pyramidal neurons. Average filters are shown in blue. Gray areas indicate one standard deviation across neurons. **(F)** GLM linear filter *κ*_GLM_(*t*) (blue). For comparison, a rescaled version of the GIF filter *κ*(*t*) is shown in red. Inset: same data shown on semi-logarithmic scales. To quantify the slow *τ*_slow_ (panel *B*, inset) and the fast *τ*_fast_ timescale of *κ*_GLM_(*t*), we performed a double exponential fit (not shown for clarity) of *κ*_GLM_(*t*). **(G)** GLM spike-history filter *h*_GLM_(*t*) (blue). For comparison, a rescaled version of the effective GIF adaptation filer *h*(*t*) (c.f., Eq 7) is shown in red. **(H)** Distribution of the GLM parameter *E*_0_ extracted from ten L5 pyramidal neurons.

After AEC, the *in vitro* recordings acquired from ten different L5 pyramidal neurons (Fig 8A) were used to perform GIF model parameter extraction (Fig 8B–8E). All of the extracted parameters were consistent with the ones previously obtained by fitting the GIF model on *in vitro* recordings from L5 pyramidal neurons responding to a mean-modulated input [31]. The parameters describing the passive properties of the membrane (i.e., the resting membrane potential *E*_L_, the membrane timescale *τ*_m_ and the input resistance *R*) revealed the presence of cell-to-cell variability (Fig 8B and 8E) and were on average consistent with previous results obtained using standard characterization protocols based on current-step injections [48]. Our characterization approach further showed that, in L5 Pyr neurons, spike-frequency adaptation is mediated by a long-lasting adaptation current featuring a power-law decay (Fig 8C; see also ref. [31]), which possibly results from the combined action of multiple channels operating on different timescales [49]. In standard protocols for single-neuron characterization, spike-triggered currents are generally assessed indirectly by measuring the spike after-hyperpolarization (AHP) induced by an artificial current pulse designed to evoke one or more action potentials (see, e.g., refs. [49, 50]). Importantly, with our characterization method the magnitude and the time-course of adaptation currents mediating AHPs can be measured simultaneously along with the other GIF model parameters, while neurons are processing *in vivo*-like inputs. Finally, our characterization protocol showed the presence of large firing threshold movements triggered by the emission of action potentials and lasting for several hundreds of milliseconds (Fig 8D). The dynamical properties of the firing threshold have been previously shown to be cell-type specific [30, 51] and functionally relevant [52], but are generally not considered in standard characterization protocols.

To allow for a comparison, we also used the *in vitro* recordings to perform GLM parameter extraction (Fig 8F–8H, see Materials and Methods). Confirming the results reported in the previous section, the effective spike-history filter *h*(*t*) of the GIF model obtained by combining the spike-triggered current *η*(*t*) and threshold movement *γ*(*t*) was in good agreement with the GLM spike-history filter *h*_GLM_(*t*) (Fig 8G). The linear filters *κ*_GLM_(*t*) and *κ*(*t*) were also in good agreement (Fig 8B and 8F, *τ*_m_ = 20.9 ms, s.d. 6.5 ms GIF; *τ*_slow_ = 22.5 ms, s.d. 3.0 ms GLM). However, the large values observed in the first two bins of *κ*_GLM_(*t*) indicated the presence of a second rapid component (*τ*_fast_ = 1.9 ms, s.d. 0.5 ms), which is neglected in the GIF model (Fig 8F, inset).

**Fig 9:**
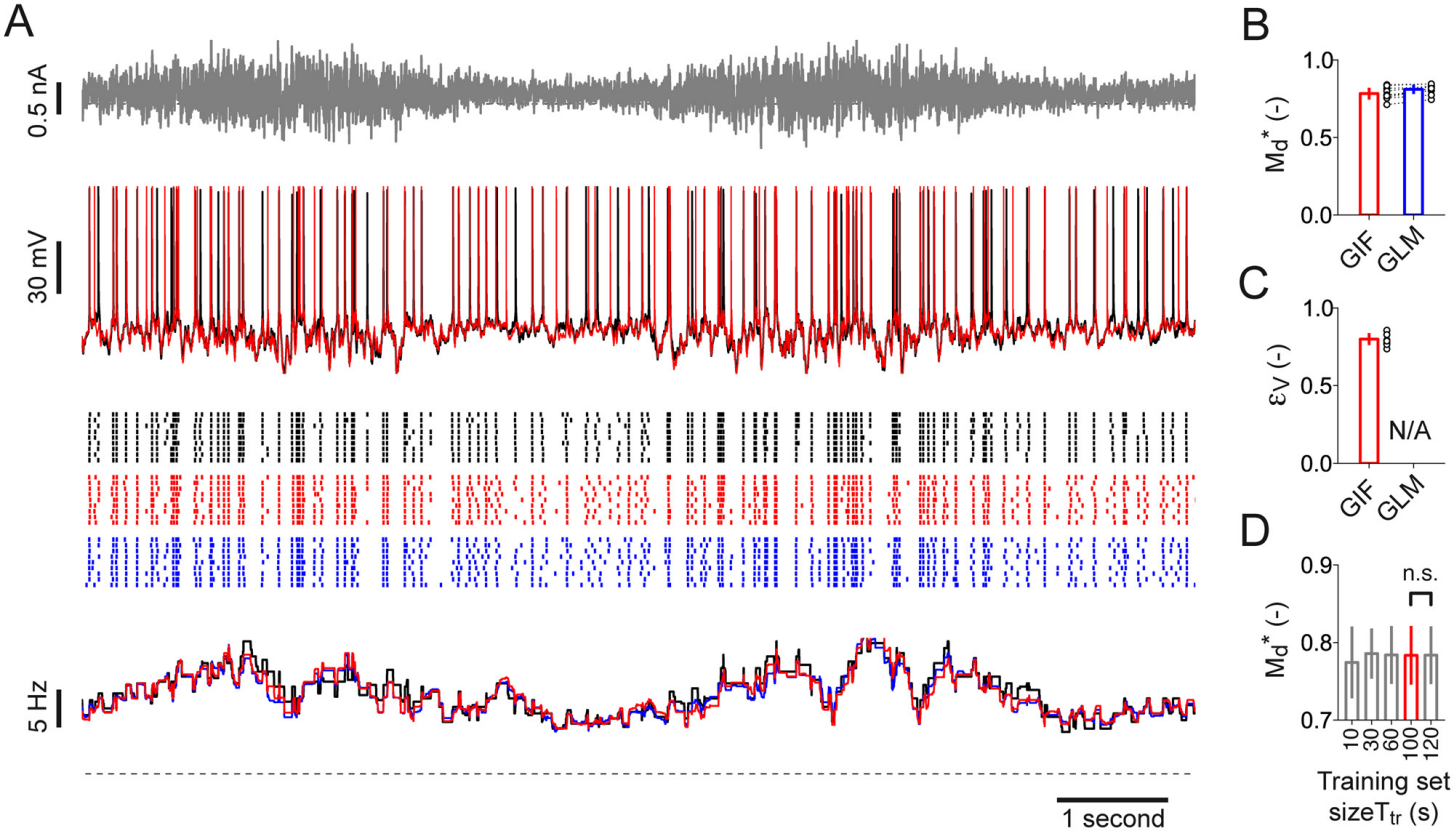
Testing the protocol for high-throughput single-neuron characterization on *in vitro* patch-clamp recordings: GIF model validation. **(A)** Input current *I*_test_(*t*) (top, gray) used for model validation, typical L5 pyramidal neuron response evoked by a single current injection (middle, black) spiking activity observed in response to nine repetitive injections of the same input (bottom, black raster) and PSTH constructed by averaging the nine spike trains within rectangular windows of 500 ms (bottom, black line). GIF model and GLM predictions are shown in red and blue, respectively. **(B)-(D)** Summary data for the performance of the GIF model and the GLM in predicting the responses of ten L5 pyramidal neurons. Each couple of open circles indicates the performance on an individual cell. Error plots indicate the mean and one standard deviation across neurons. **(B)** Spike-timing prediction as quantified by $M_{d}^{*}$ with precision Δ = 4 ms. **(C)** Mean prediction error *ϵ*_V_ on subthreshold membrane potential fluctuations. The GLM does not explicitly model the subthreshold membrane potential dynamics and is therefore not applicable (N/A). **(D)** GIF model spike-timing prediction ($M_{d}^{*}$, with precision Δ = 4 ms) as a function of the training set size used for parameter extraction. Increasing the duration of the training set from 100 s to 120 s does not improve the GIF model predictive power ($M_{d}^{*}$ = 0.79, s.d. 0.04, *T*_tr_ = 100 s; $M_{d}^{*}$ = 0.79, s.d. 0.04, *T*_tr_ = 120 s; *n* = 10, paired Student *t*-test, *t*_9_ = 0.25, *p* = 0.8; n.s. > 0.05).

We tested the predictive power of both the GIF model and the GLM on a new set of recordings (*test set*) in which a test current *I*_test_(*t*) was repetitively injected (Fig 9A). In terms of mere spike-timing prediction, the GIF model and the GLM achieved similar results ($M_{d}^{*}=0.79\pm 0.04$, GIF; $M_{d}^{*}=0.81\pm 0.04$, GLM; Fig 9B). Moreover, the GIF model, but not the GLM, could predict the subthreshold response of real neurons with an RMSE of 3.6 mV, s.d. 0.5 mV (variance explained *ϵ*_V_ = 80.1 %, s.d. 4.1%; Fig 9C). These results indicate that the difference observed between the linear filters *κ*(*t*) and *κ*_GLM_(*t*) does not have a major impact on the predictive power of the models.

Finally, comparing the predictive power of different GIF models with parameters extracted from five training sets of different durations (*T*_tr_ = 10, 30, 60, 100 and 120 s; Fig 9D) confirmed that 100 seconds of intracellular recordings are sufficient to accurately constrain the GIF model parameters. We conclude that our protocol for GIF model parameter extraction is suitable for high-throughput single-neuron characterization.

## Discussion

The intrinsic dynamics of individual neurons strongly differ between cell types and brain areas [53]. This heterogeneity is increasingly considered as a critical feature of the brain and not as a consequence of biological imprecision [54, 55]. Taking into account single-neuron variability may be crucial to understand how neural systems support computation. In the near future, automated electrophysiology will likely make available increasingly large amounts of data. Due to their inherent complexity (and their high dimensionality), raw data from patch-clamp recordings are difficult to interpret and cannot be directly clustered to identify electrophysiological types. GIF models are currently employed by computational neuroscientists mainly to investigate the emergent properties of neural networks. Here, we showed that these models also comprise a powerful tool to cast the information provided by voltage recordings into small sets of model parameters that can be easily interpreted and compared.

More precisely, we demonstrated that the fitting procedure for GIF models we recently introduced [31, 37] (Figs 1 and 2) is suitable for high-throughput analysis of intracellular patch-clamp recordings. Using an artificial dataset generated by the model itself, we first established that GIF model parameter extraction and validation can be accomplished in around five minutes given a limited amount of intracellular recordings (Fig 3). Based on these results, we then designed a protocol for the characterization of the electrical activity of single neurons (Fig 4). On the experimental side, the protocol relies on *in vitro* injections of rapidly fluctuating currents. To compensate for the artifact known to occur while delivering inputs through the recording electrode, we propose the use of Active Electrode Compensation [32, 33] (Fig 7). In AEC, estimating the electrode properties is a potentially time-consuming procedure. For this reason, in our protocol, artifacts resulting from the voltage drop across the patch-clamp electrode are removed only after the complete acquisition of the dataset used for parameter extraction (Fig 4). We tested the protocol for high-throughput single-neuron characterization using both *in silico* data (Figs 5 and 6) as well as *in vitro* recordings obtained with conventional (i.e., manual) patch-clamping (Figs 8 and 9). In both cases the results confirmed our conclusion drawn from the analysis of artificial data generated by the GIF model itself (Fig 3)X namely that a GIF model with parameters extracted from a training set with size larger than 30 seconds accurately predicts both the subthreshold and the spiking response evoked by a new input. Considering that long current-clamp recordings are generally affected by low-frequency artifacts such as drifts in resting membrane potential, access resistance and average firing rate, it seems unlikely that a training set whose size prohibits rapid characterization would improve accuracy. Intriguingly, we found that the GIF model achieves almost identical performances in predicting *in silico* and *in vitro* data (Figs 6 and 9), indicating that detailed biophysical models could be used in the future to guide the improvement of simplified spiking models. Analyzing the performance of the GIF model in response to dendritic inputs goes beyond the scope of this study. However, as demonstrated by a recent study [56], the mathematical framework discussed here is flexible and can in principle be extended to account for dendritic current injections.

Considering the time required to automatically select a target neuron and form a gigaohm seal, our results demonstrate that, if combined with emergent technologies for automatic patch-clamping, the mathematical tools discussed in this study could be used to implement a high-throughput pipeline performing single-neuron characterization in around ten minutes. Importantly, all the computations in the protocol can be executed *on the fly*, while electrophysiological recordings are being performed. Consequently, the model performance in predicting the spiking activity (i.e., $M_{\text{d}}^{*}$) and the subthreshold voltage dynamics (i.e., *ϵ*_V_) could be used for online monitoring and quality control, possibly allowing for automated detection of experimental problems. Online characterization and identification of neurons may also prove useful in more detailed high-throughput characterization of neuronal cell types currently being set up in the context of several large-scale brain initiatives as this would allow for *on the fly* implementation of cell-specific stimulus sets. Similarly, online identification could be useful in manual patch-clamp experiments whose aim is not to perform high-throughput single-neuron characterization, but is to study other neuronal properties (e.g., the dynamics of specific ion-channels under pharmacology, the effect of neuromodulators on the response properties of neurons, connectivity, short-term and spike-timing dependent plasticity). These experiments generally start with a brief set of current injections (e.g., current-steps) aimed at identifying some basic features of the neuronal dynamics (e.g., passive membrane properties, firing patterns). Given its short duration and its limited requirements in terms of computing power, our protocol could provide an alternative in these situations.

To allow for a comparison, both *in silico* and *in vitro* recordings were also fitted with a GLM [35, 36]. Despite the fact that GLMs are more flexible than GIF models, we found that, in terms of mere spike timing prediction, the two models achieved similar performance (Figs 6 and 9). This result can be understood by noting that the *nonparametric* filter *κ*_GLM_(*t*) extracted with the GLM fitting procedure is well approximated by the exponential filter *κ*(*t*) of the GIF model. GLMs are typically considered as statistical models for spike trains and their parameters are only loosely related to biophysical cell properties. The reason for this is that GLM parameter extraction entirely relies on the likelihood maximization of the spiking data. If on one hand this fact constitutes a big advantage in case of (multi-electrode) extracellular recordings [22, 36], the standard GLM framework is less appropriate for whole-cell current-clamp data. In contrast to GIF models, GLMs do not explicitly model the membrane potential dynamics, do not exploit all the information available in intracellular recordings and, consequently, are unable to predict the subthreshold activity of single neurons. Moreover, compared to GLMs, we found that parameter extraction for GIF models is faster.

A voltage-dependent plasticity rule has recently been proposed [57] in which the subthreshold dynamics of the membrane potential plays a crucial role in explaining a large variety of experimental results obtained using different induction protocols for long-term potentiation (or depression). Among others, this finding highlights the need of spiking neuron models that accurately capture the subthreshold membrane potential dynamics. The GIF model accounts for spike-dependent adaptation using two distinct filters: a spike-triggered current *η*(*t*) and a spike-triggered movement of the firing threshold *γ*(*t*). At first glance, having two spike-dependent processes might seem redundant and unnecessary. However, while the firing threshold only affects spike probability, adaptation currents also alter the dynamics of the subthreshold membrane potential. This explains why the correct distinction between these two forms of adaptation is key to correctly predict the subthreshold response of single neurons. Supporting this claim, a reduced GIF model, in which the two processes mediating spike-frequency adaptation are combined into a single effective filter *h*(*t*) (Eq 7), has been shown to systematically overestimate the membrane potential [31].

Since GLM parameter extraction entirely relies on spiking data (see Materials and Methods), the linear filter *κ*_GLM_(*t*) also includes the effects of all biophysical processes that affect spike emission without altering the subthreshold membrane potential. In particular, the filter *κ*_GLM_(*t*), but not the filter *κ*(*t*) of the GIF model, is expected to capture a potential coupling between subthreshold voltage and firing threshold [58, 59]. Possibly explaining the difference we found between *κ*_GLM_(*t*) and *κ*(*t*) (Fig 8F), both direct [60] and indirect [52] experimental evidence has been provided that such a coupling exists in cortical pyramidal neurons. Extending the GIF model to account for a coupling between membrane potential and firing threshold is beyond the scope of this study and will be presented in a separate publication. It is however worth noting that the threshold equation of the GIF model can be easily augmented as follows:

$$\begin{array}{c} V_{T}\left(t\right)=V_{T}^{*}+\underset{{\hat{t}}_{\text{j}}<t}{\sum }\gamma \left(t\gamma (t-{\hat{t}}_{j})-{\hat{t}}_{j}\right)+\overset{t}{\underset{{\hat{t}}_{\text{last}}}{\int }}\kappa_{\theta }\left(s\right)V\left(t-s\right)ds, \end{array}$$ (9)

with *κ*_*θ*_(*t*) being an arbitrarily shaped filter that, with a straightforward extension of the maximum likelihood method used in Step 3 (see Fig 2, *Step 3*), could be extracted from intracellular recordings.

In contrast to the GIF model, popular point-neuron models like the adaptive exponential integrate-and-fire (ADEX, [61]) or the adaptive quadratic integrate-and-fire (AQIF, [62]) feature a subthreshold adaptation current *w*(*t*) governed by the following differential equation

$$\begin{array}{c} \tau_{w}\dot{w}=-w+a\left(V-E_{L}\right). \end{array}$$ (10)

Extending the GIF model with Eq 10 would relax the assumption of having a single exponential membrane filter *κ*(*t*) and, depending on the parameter choice, the subthreshold dynamics of the resulting model could account for two-timescale decay or resonance [30]. In ADEX and the AQIF model, this current has been shown to play an important role in explaining the variety of firing patterns emitted by single neurons in response to a step of current [63, 64]. In the GIF model, the lack of subthreshold adaptation is, at least partially, compensated by the fact that the spike-triggered current is not assumed to be exponential, but can have an arbitrary shape. For example, the GIF model can capture the *resonate-and-fire* behavior by means of a biphasic spike-triggered current. Such a current hyperpolarizes the membrane during the first milliseconds and then rapidly becomes positive, thereby favoring the emission of spikes with a particular interspike interval [37]. Our results suggest that, while increasing the complexity, extending a GIF model with a subthreshold current *w*(*t*) does not significantly improve the model’s performance in predicting the activity of the three main neuronal types of the mouse barrel cortex [30]. However, this might not hold true for neurons in other brain regions or in the case of more sophisticated stimulation paradigms. Performing parameter extraction with a GIF model extended with Eq 10 is possible. Once the timescale *τ*_*w*_ is known, performing a least-square regression similar to Eq 17 is indeed sufficient to recover all the other parameters. Extended GIF model parameter extraction can therefore be performed by iterating on *τ*_*w*_ and looking for the timescale that minimizes the sum of squared errors on the voltage derivative. Since line-search (i.e., brute-force) algorithms can be efficiently executed using parallel computing, extending a GIF model with a subthreshold adaptation current does not necessarily imply a dramatic increase of the CPU time required for parameter extraction.

In the field of computational neuroscience, the last years have been characterized by the announcements of several large-scale projects aimed to build realistic models of the electrical activity of entire brains [8, 9, 65–67]. To achieve this ambitious goal, it is of crucial importance to characterize and model the diversity amongst the brain’s fundamental building blocks: the single neurons. Here, we demonstrate that, if combined with automatic patch-clamp recordings, a fitting technique for GIF models, which we recently introduced [31, 37], can be used to build a pipeline for high-throughput single-neuron characterization and modeling.

## Materials and Methods

### Ethics Statement

All procedures in this study were conducted in conformity with the Swiss Welfare Act and the Swiss National Institutional Guidelines on Animal Experimentation for the ethical use of animals. The Swiss Cantonal Veterinary Office approved the project following an ethical review by the State Committee for Animal Experimentation.

### Electrophysiological recordings

*In vitro* electrophysiological recordings were performed on 300 *μ*m thick parasagittal acute slices from the right hemispheres of male P13–15 C57Bl/6J mouse brains, which were quickly dissected and sliced (HR2 vibratome, Sigmann Elektronik, Germany) in ice-cold artificial cerebrospinal fluid (ACSF) (in mM: NaCl 124, KCl 2.5, MgCl_2_ 10, NaH_2_PO_4_ 1.25, CaCl_2_ 0.5, D-(+)-Glucose 25, NaHC0_3_ 25; pH 7.3, s.d. 0.1, aerated with 95% O_2_, 5% CO_2_), followed by a 15 minute incubation at 34°C in standard ACSF (in mM: NaCl 124, KCl 2.5, MgCl_2_ 1, NaH_2_PO_4_ 1.25, CaCl_2_ 2, D-(+)-Glucose 25, NaHC0_3_ 25; pH 7.4, aerated with 95% O_2_, 5% CO_2_), equally used as bath solution. Cells were visualized using infrared differential interference contrast video microscopy (VX55 camera, Till Photonics, Germany and BX51WI microscope, Olympus, Japan). Somatic whole-cell current clamp recordings of layer 5 pyramidal cells in the primary somatosensory cortex were performed at 32, s.d. 1°C with an Axon Multiclamp 700B Amplifier (Molecular Devices, USA) using 6.5–7.5 MΩ borosilicate pipettes, containing (in mM): K^+^–gluconate 110, KCl 10, ATP–Mg^2+^ 4, ${\text{Na}}_{2}^{+}$–phosphocreatine 10, GTP–Na^+^ 0.3, HEPES 10, biocytin 5 mg/ml; pH 7.3, 300 mOsm). To ensure intact axonal and dendritic arborisation, recordings were conducted in slices cut parallel to the apical dendrites.

Data were acquired at Δ*T* ^−1^ = 20 kHz using an ITC-18 digitising board (InstruTECH, USA) controlled by a custom-written software module operating within IGOR Pro (Wavemetrics, USA). Voltage signals were low-pass filtered (Bessel, 10 kHz) and not corrected for the liquid junction potential. Only cells with an access resistance < 25MΩ (20.2, s.d. 3.2 MΩ, *n* = 10), which was compensated throughout the recording, and a drift in the resting membrane potential < 2.5 mV (1.2 mV, s.d. 0.8 mV, *n* = 10) between the start and the end of the recording were retained for further analysis.

### *In silico* recordings: multi-compartmental model simulations

*In silico* recordings were performed by simulating a multi-compartmental model of aN L5b pyramidal neuron [14]. The model was obtained from Model DB (accession number 139653) and all simulations were performed in Neuron [68]. Similar to the *in vitro* experiments, input currents *I*(*t*) were generated according to Eqs 5–6 (with sampling frequency Δ*T* ^−1^ = 20 kHz) and were delivered at the somatic compartment. To obtain an average firing rate fluctuating between 7 and 13 Hz, the input parameters were set to *I*_0_ = 520 pA, *σ*_0_ = 320 pA and Δ*σ* = 0.5. To reproduce spike timing variability between responses to repetitive injections of the same current *I*(*t*), a source of noise was included in the model by adding a zero-mean white-noise signal *ξ*_w.n._(*t*) to *I*(*t*). In order to capture the autocorrelation function between spike trains recorded *in vitro* in response to different repetitions of the test set current, the magnitude of the noise was set to $\sqrt{\langle \xi_{\text{w}.\text{n}.}{\left(t\right)}^{2}\rangle }=$ 160 pA. The same amount of noise was also used to generate the training dataset. GIF model and GLM parameter extraction were performed by treating the noise current *ξ*_w.n._(*t*) as being unknown.

### Data preprocessing: Active Electrode Compensation

All the *in vitro* recordings included in this study were preprocessed using AEC [32] according to the following four-step procedure [31, 33].

**Step 1**: Shortly before the acquisition of the *training dataset* (see Fig 4), we recorded the intracellular response *V*_sub_(*t*) evoked by the injection of a short subthreshold current *I*_sub_(*t*). The input was generated according to Eq 5 with parameters *I*_0_ = 0 pA, *σ*(*t*) = 75 pA and *τ* = 3 ms and evoked small-amplitude subthreshold fluctuations around the resting potential. With this parameter choice, the standard deviation of *V*_sub_(*t*) was around 2–3 mV.

**Step 2**: We then estimated the optimal linear filter *κ*_opt_(*t*) between the subthreshold input *I*_sub_(*t*) and the recorded signal *V*_sub_(*t*) (Fig 7B). To reduce computing time, *κ*_opt_(*t*) was defined over a finite interval [0,200ms] as

$$\begin{array}{c} \kappa_{\text{opt}}\left(t\right)=\sum_{m=1}^{M}b_{m}f^{\left(m\right)}\left(t\right), \end{array}$$ (11)

with {*f* ^(*m*)^(*t*)} being a set of M = 202 rectangular basis functions of linearly increasing width. The parameters *b* = [*b*_1_, …, *b*_*M*_] determining the shape of *κ*_opt_(*t*) were then estimated by solving the following multilinear regression:

$$\begin{array}{c} b={\left(Z^{T}Z\right)}^{-1}Z^{T}V, \end{array}$$ (12)

where, using the discrete-time notation *x*_*t*_ = *x*(*t* · Δ*T*) and by removing the subscripts *sub* for clarity, *V* is a vector whose *t*-th element is given by the membrane potential *V*_*t*_ = *V*(*t*Δ*T*) and *Z* is a matrix made of vectors $z_{t}^{T}$ defined as:

$$\begin{array}{c} z_{t}^{T}=[\;\sum_{s=0}^{t}f_{\text{s}}^{\left(1\right)}I_{t-s}\Delta T,\;\ldots \;,\sum_{s=0}^{t}f_{\text{s}}^{\left(M\right)}I_{t-s}\Delta T\;], \end{array}$$ (13)

with *I*_*t*_ = *I*(*t*Δ*T*).

**Step 3**: An exponential function *f*(*t*;*a*_1_, *a*_2_) = *a*_1_exp(−*t*/*a*_2_) was then fitted to the tail of *κ*_opt_(*t*) by minimizing the error $E(a_{1},a_{2})=\int_{T_{\text{min}}}^{\infty }{\left(\kappa_{\text{opt}}(s)-f(s;a_{1},a_{2})\right)}^{2}ds$ (Fig 7B). In AEC, the electrode is assumed to operate on fast timescales (< 1 ms) and the slow decay in *κ*_opt_(*t*) is attributed to the cell. For this reason the fit was performed with *T*_min_ = 5 ms, and the electrode filter was estimated as

$$\begin{array}{c} \kappa_{\text{e}}\left(t\right)=\kappa_{\text{opt}}\left(t\right)-f\left(t;{\hat{a}}_{1},{\hat{a}}_{2}\right), \end{array}$$ (14)

with ${\hat{a}}_{1}$ and ${\hat{a}}_{2}$ being the optimal parameters minimizing *E*(*a*_1_, *a*_2_). To improve accuracy, Steps 2 and 3 were repeated 15 times by resampling from the available data and the final electrode filter used for AEC was obtained by averaging the results across repetitions (Fig 7C). Alternatively, the electrode filter *κ*_e_(*t*) can be extracted from *κ*_opt_(*t*) by considering that also the net current flowing through the cell membrane is affected by the electrode properties (see ref. [32]).

**Step 4**: Finally, for all subsequent current-clamp injections, the membrane potential *V*_data_(*t*) was estimated as follows (Fig 7A and 7D):

$$\begin{array}{c} V_{\text{data}}\left(t\right)=V_{\text{rec}}\left(t\right)-\int_{0}^{\infty }\kappa_{\text{e}}\left(s\right)I_{\text{ext}}\left(t-s\right)ds, \end{array}$$ (15)

where *I*_ext_(*t*) is the injected current, *V*_rec_(*t*) is the recorded signal and the convolution integral on the right-hand side of Eq 15 approximates the voltage drop *V*_e_(*t*) across the electrode.

Expanding *κ*_opt_(*t*) in rectangular basis functions drastically reduces the computing time required in Step 2. Overall, Steps 1–3 were performed in around 62 seconds and can in principle be executed while the *training set* is being acquired. Step 4 requires less than 1 second and can be performed after training set collection without compromising high-throughput (Fig 4). Since in our protocol model validation only relies on spike-timing prediction, AEC only has to be applied to the training dataset. Here, in order to asses the prediction error on the subthreshold membrane potential, we also performed AEC on all test set recordings. In order to evaluate the quality of the recordings, our protocol for high-throughput single-neuron characterization (Fig 4) could in principle be extended by repeating Steps 1–3 after complete acquisition of the *test set*. These additional data could indeed be used to verify whether the electrode properties (i.e., the access resistance, the electrode timescale and, more generally, the electrode filter *κ*_e_(*t*)) were satisfactory and sufficiently stable during the experiment (see ref. [31]).

### GIF model parameter extraction

Given the intracellular membrane potential *V*_data_(*t*) measured at a sampling frequency Δ*T* ^−1^ in response to a known input current *I*_tr_(*t*), as well as the spike times $\left\{{\hat{t}}_{j}\right\}$ defined as instants at which *V*_data_(*t*) crosses 0 mV from below, all the GIF model parameters are extracted following a three-step procedure [31, 37] (Fig 2).

**Step 1**: The absolute refractory period *T*_ref_ is fixed to an arbitrary value and the voltage reset is estimated by the average membrane potential recorded *T*_ref_ milliseconds after a spike $V_{\text{reset}}={\langle V_{\text{data}}({\hat{t}}_{j}+T_{\text{ref}})\rangle }_{j}$. Since absolute refractoriness can be captured by a spike-triggered movement of the firing threshold, the particular choice of *T*_ref_ is not crucial and the only constraint is given by the shortest interspike interval in the dataset. Here, for L5 pyramidal neurons, we set the refractory period to *T*_ref_ = 4 ms.

**Step 2**: The parameters determining the subthreshold dynamics of the membrane potential are extracted. To allow convex optimization, the spike-triggered current *η*(*t*) is expanded as a linear combination of basis functions [22]:

$$\begin{array}{c} \eta \left(t\right)=\sum_{k=1}^{K}\eta_{k}f^{\left(k\right)}\left(t\right), \end{array}$$ (16)

where {*η*_*k*_} is a set of parameters controlling the time course of *η*(*t*). The parameters $\theta_{\text{sub}}^{T}=C^{-1}\cdot [g_{\text{L}},E_{\text{L}}g_{\text{L}},\eta_{1},...,\eta_{K},1]$ are then extracted by minimizing the sum of squared errors between the observed voltage derivative ${\dot{V}}_{\text{data}}$ and that of the model (i.e., Eq 1). Since all subthreshold parameters *θ*_sub_ act linearly on the observables, this optimization problem can be efficiently solved by computing the following multilinear regression [12, 22]:

$$\begin{array}{c} {\hat{\theta }}_{\text{sub}}={\left(X^{T}X\right)}^{-1}X^{T}{\dot{V}}_{\text{data}}, \end{array}$$ (17)

where *X* is a matrix whose rows are given by the vectors

$$\begin{array}{c} x_{t}^{T}=[-V_{\text{data}}\left(t\right),1,-\sum_{j}f^{\left(1\right)}\left(t-T_{\text{ref}}-{\hat{t}}_{j}\right),\;\ldots \;,-\sum_{j}f^{\left(K\right)}\left(t-T_{\text{ref}}-{\hat{t}}_{j}\right),I\left(t\right)], \end{array}$$ (18)

and ${\dot{V}}_{\text{data}}$ is a column-vector containing the voltage first-order derivative estimated by finite differences ${\dot{V}}_{\text{data}}(t)=\left(V_{\text{data}}(t+\Delta T)-V_{\text{data}}(t)\right)/\Delta T$. Since the GIF model does not capture the subthreshold dynamics during spike initiation, all the data points close to action potentials $\left\{t|t\in [{\hat{t}}_{j}-5\text{ms};{\hat{t}}_{j}+T_{\text{ref}}]\right\}$ are excluded from the regression.

In principle, the residuals of this multilinear regression could provide some additional information about the quality of the recordings and could therefore be used for online quality control. This is true especially in cases where a large number of experiments are repeated in similar cell types and under similar conditions. In such a situation, one can indeed evaluate the results with respect to the distribution of errors obtained in previous experiments. Although the magnitude of high-frequency noise in patch-clamp recordings is generally low, this metric might however depend on the experimental sampling frequency. Overall, to limit the impact of high-frequency noise, it is generally safer to assess the model performance by computing the residuals on *V*_data_ (c.f., Eq 27), rather than on ${\dot{V}}_{\text{data}}$.

**Step 3**: The parameters defining the dynamics of the firing threshold are extracted. To determine the functional shape of the spike-triggered movement of the firing threshold, we first expand *γ*(*t*) as a sum of basis functions:

$$\begin{array}{c} \gamma \left(t\right)=\sum_{p=1}^{P}\gamma_{p}f^{\left(p\right)}\left(t\right). \end{array}$$ (19)

Given the parameters obtained in the first two steps and the spike times observed in the experiment, the subthreshold membrane potential ${\hat{V}}_{\text{model}}(t)$ is then computed by numerical integration of Eq 1. Without loss of flexibility, the parameter *λ*_0_ is fixed to 1 Hz and all threshold parameters $\theta_{\text{th}}^{T}=\Delta V^{-1}\cdot [1,V_{\text{T}}^{*},\gamma_{1},...,\gamma_{P}]$ are finally extracted by maximizing the log-likelihood of the experimental spike-train [35, 36, 69]:

$$\begin{array}{c} {\hat{\theta }}_{\text{th}}=\underset{\theta_{\text{th}}}{\mathrm{argmax}}\{\sum_{t\in \{{\hat{t}}_{j}\}}y_{t}^{T}\theta_{\text{th}}-\Delta T\cdot \sum_{t\in \Omega }\exp(y_{t}^{T}\theta_{\text{th}})\}, \end{array}$$ (20)

where $\Omega =\{t|t\notin [{\hat{t}}_{j},{\hat{t}}_{j}+T_{\text{ref}}\;]\}$ is a set that excludes all the data points falling in the absolute refractory periods and the vectors $y_{t}^{T}$ are defined as

$$\begin{array}{c} y_{t}^{T}=[{\hat{V}}_{\text{model}}\left(t\right),-1,-\sum_{j}f^{\left(1\right)}\left(t-T_{\text{ref}}-{\hat{t}}_{j}\right),\;\ldots \;,-\sum_{j}f^{\left(P\right)}\left(t-T_{\text{ref}}-{\hat{t}}_{j}\right)]. \end{array}$$ (21)

With the exponential function in Eq 2, the log-likelihood to maximize is a convex function of *θ*_th_[41] and both its gradient and Hessian can be computed analytically. Consequently, the optimization problem of Eq 20 can be efficiently solved using the Newton-Raphson (gradient ascent) method. Alternatively, Step 3 can be performed using the recorded potential *V*_data_(*t*) instead of ${\hat{V}}_{\text{model}}(t)$ in Eq 21. Since small inaccuracies in Step 2 can be compensated in *Step 3*, performing the fit using ${\hat{V}}_{\text{model}}(t)$ generally improves spike-timing prediction.

In order to avoid numerical problems resulting from the inappropriate choice of the initial condition *θ*_th,0_ used in Step 3, it is convenient to first find the optimal threshold parameters ${\hat{V}}_{\text{T}}^{*}$ and $\hat{\Delta V}$ of a reduced GIF model in which the firing threshold is constant (i.e., $V_{\text{T}}=V_{\text{T}}^{*}$). For that, a first gradient ascent is performed with initial conditions Δ*V*_0_ = 50 mV and $V_{T,0}^{*}=-\Delta V_{0}\cdot \text{log}\;\bar{r}$, where $\bar{r}$ denotes the average firing rate in the data. Then, a second gradient ascent is performed on the log-likelihood of the full model using $\theta_{\text{th,0}}={\hat{\Delta V}}^{-1}\cdot [1,{\hat{V}}_{\text{T}}^{*},0,...,0]$ as initial condition.

### Generalized Linear Model

All GIF model performance included in this study are compared against the ones of a standard GLM [35, 36]. In the GLM, spikes are emitted stochastically according to the following conditional intensity

$$\lambda_{\text{GLM}}(t|I,\left\{{\hat{t}}_{j}\right\})=\lambda_{0}\cdot \text{exp}\left(E_{0}+\overset{t}{\underset{0}{\int }}\kappa_{\text{GLM}}\left(s\right)I\left(t-s\right)ds+\underset{{\hat{t}}_{j}<t}{\sum }h_{\text{GLM}}\left(t-{\hat{t}}_{j}\right)\right),$$ (22)

with *λ*_0_ = 1 Hz. In the GLM, the linear filter *κ*_GLM_(*t*) is not assumed to be exponential but is extracted from experimental data through linear expansion in rectangular basis functions. Moreover, the GLM accounts for spike-history effects with a unique filter *h*_GLM_(*t*). The GLM also differs from the GIF model because it has neither an absolute refractory period nor an explicit reset after the emission of a spike. To obtain a fair comparison between the two models, the filter *h*_GLM_(*t*) was expanded using the same basis functions as used for *γ*(*t*) in the GIF and the number of basis functions used for *κ*_GLM_(*t*) was such that, in total, the two models had the same number of parameters. Given the input current *I*(*t*) and the observed spike train *S*_data_(*t*), GLM parameters *θ*_GLM_ = {*E*_0_, *κ*_GLM_(*t*), *h*_GLM_(*t*)} were extracted with standard methods [35, 36] by maximizing the model log-likelihood *L*(*θ*_GLM_) = log*p*(*S*_data_∣*I*, *θ*_GLM_) using $\theta_{\text{GLM,0}}=\{\text{log}\bar{r},0,0\}$ as initial condition. Importantly, the GLM fitting procedure does not exploit the information available in the subthreshold membrane potential fluctuations and all of its parameters are extracted using the maximum likelihood approach. This explains why fitting a GLM requires more CPU time than fitting a GIF model.

### Similarity measure $M_{d}^{*}$ between sets of spike trains

To quantify the model performance in predicting spikes, we used the normalized, bias-corrected metrics $M_{d}^{*}$ [34]. $M_{d}^{*}$ relies on a measure of the distance between the experimental and the predicted spike-emission probability, which are in turn inferred from the responses to a limited number of repetitive current injections. Importantly, $M_{d}^{*}$ resolves the *small sample* bias known to affect most of the similarity measures when the number of available spike trains is small. Also, in contrast to previous measures based on naive pairwise comparisons (e.g., the Γ coincidence factor used in ref. [70]), $M_{d}^{*}$ does not suffer from the so-called *deterministic bias* known to favor noise-free models and is therefore well suited for the evaluation of stochastic spiking models [34]. Moreover, in contrast to many other correlation-based measures, $M_{d}^{*}$ is sensitive to the accuracy with which both the shape and the amplitude of the spike probability is predicted.

Given a small set of experimental spiketrains $S_{i}^{\text{(d)}}=\sum_{f}\delta (t-{\hat{t}}_{f})$ recorded in response to *N*_d_ repetitive injections *i* = 1, …, *N*_d_ of the same input current *I*_test_(*t*), as well as a large set of spike trains $S_{j}^{\text{(m)}}=\sum_{f}\delta (t-{\hat{t}}_{f})$ predicted by *N*_m_ repetitive simulations *j* = 1, …, *N*_m_ of a stochastic model, the similarity $M_{d}^{*}$ between the two sets of spike trains is defined as [34]:

$$\begin{array}{c} M_{d}^{*}=\frac{2\cdot \langle \nu_{\text{d}},\nu_{\text{m}}\rangle }{\frac{2}{N_{\text{d}}\left(N_{\text{d}}-1\right)}\sum_{i=1}^{N_{\text{d}}}\sum_{i^{'}=i+1}^{N_{\text{d}}}\langle S_{i}^{\text{(d)}},S_{i^{'}}^{\text{(d)}}\rangle +\langle \nu_{\text{m}},\nu_{\text{m}}\rangle }, \end{array}$$ (23)

where $S_{i}^{\text{(d)}}$ denotes the *i*-th experimental spike-train, $\nu_{\text{d}}=\frac{1}{N_{d}}\sum_{i=1}^{N_{\text{d}}}S_{i}^{\text{(d)}}$ is the average experimental response across trials (that is,i.e., the experimental peri-stimulus-time histogram (PSTH) computed with infinitesimally small bins), $\nu_{\text{m}}=\frac{1}{N_{m}}\sum_{i=1}^{N_{\text{m}}}S_{i}^{\text{(m)}}$ is the average model response and ⟨*ν*_m_, *ν*_*m*_⟩ represents its norm. Due to high-throughput requirements and experimental constraints, only a small number *N*_d_ of experimental spike-trains are available. For this reason, the norm of *ν*_d_ must be computed using an unbiased estimator (cf. first term in the denominator of Eq 23). Finally, the brackets ⟨·, ·⟩ denote the inner product used to quantify the distance between two spike trains [34]:

$$\begin{array}{c} \left\langle S_{i},S_{j}\right\rangle =\int_{0}^{T}\int_{-\infty }^{\infty }\int_{-\infty }^{\infty }K\left(s,s^{'}\right)S_{i}\left(t-s\right)S_{j}\left(t-s^{'}\right)dsds^{'}dt, \end{array}$$ (24)

where *K*(*s*, *s*′) is a two-dimensional kernel defining the degree of coincidence between two spikes occurred at times *s* and *s*′.

While different windows *K*(*s*, *s*′) may be used, the Kistler coincidence kernel *K*(*s*, *s*′) = *δ*(*s*′) · Θ(*s*+Δ) · Θ(−*s*+Δ) was chosen with Δ = 4 ms as in refs.[30, 31]. With this particular choice, the inner product ⟨*S*_*i*_, *S*_*j*_⟩ equals the number of spikes in *S*_*i*_ that fell ±Δ ms apart to one of the spikes in *S*_*j*_ and, consequently, $M_{d}^{*}$ becomes:

$$\begin{array}{c} M_{d}^{*}=\frac{2n_{\text{dm}}}{n_{\text{dd}}^{*}+n_{\text{mm}}}, \end{array}$$ (25)

with *n*_dm_ being the average number of coincident spikes between data (*d*) and model (*m*), *n*_mm_ being the average number of coincident spikes computed across *N*_m_ = 500 repetitions generated by the model and $n_{\text{dd}}^{*}$ being the bias-corrected average number of coincident spikes between different experimental spike trains (i.e., the number of coincident spikes between experimental spike trains $S_{i}^{\left(d\right)}$ and $S_{j}^{\left(d\right)}$ averaged across (*i*, *j*) ∈ [1, *N*_d_] × [1, *N*_d_] with *i* ≠ *j*, see Eq 23).

### Performance evaluation

*Prediction error *ϵ*_V_ on the subthreshold response*. For each repetition *i* in the *test set*, we computed the coefficient of determination $R_{i}^{2}$ between the experimental membrane potential $V_{i}^{\text{(data)}}(t)$ and the GIF model prediction ${\hat{V}}_{i}^{\text{(model)}}(t)$ (obtained by solving Eq 1 and enforcing the spikes to occur at the same time as in the experiment):

$$\begin{array}{c} R_{i}^{2}=1-\frac{\int_{0}^{T_{\text{test}}}{\left(V_{i}^{\text{(data)}}\left(t\right)-{\hat{V}}_{i}^{\text{(model)}}\left(t\right)\right)}^{2}dt}{\int_{0}^{T_{\text{test}}}{\left(V_{i}^{\text{(data)}}\left(t\right)-{\bar{V}}_{i}^{\text{(data)}}\right)}^{2}dt}, \end{array}$$ (26)

The prediction error *ϵ*_V_ on the subthreshold response was then obtained by averaging the results from each repetition:

$$\begin{array}{c} \epsilon_{\text{V}}=\frac{1}{n_{\text{test}}}\sum_{i=1}^{n_{\text{test}}}R_{i}^{2}. \end{array}$$ (27)

*ϵ*_V_ takes values between 0 and 1 and can be interpreted as the fraction of variance of the subthreshold membrane potential fluctuations that the model was able to predict. The parameters *T*_test_ and *n*_test_ denote the duration and the number of repetitions in the *test set*, respectively.

*Prediction error *ϵ*_param_ on the GIF model parameters*. The mean error *ϵ*_param_ on the parameters *θ* extracted from artificial data is defined as

$$\begin{array}{c} \epsilon_{\text{param}}={\left\langle \frac{\Delta \theta_{i}}{|\theta_{i}|}\right\rangle }_{i}, \end{array}$$ (28)

where $\Delta \theta_{i}=|\theta_{i}-{\hat{\theta }}_{i}|$ is the *L*1-error between the estimated parameter ${\hat{\theta }}_{i}$ and the reference parameter *θ*_*i*_ (used to generate the artificial data). Overall, *ϵ*_param_ measures the absolute percentage error averaged across model parameters.

All the CPU times reported in this study were obtained using an IntelCore i7 CPU920 @ 2.67GHz with 24 GB RAM. Both GLM and GIF model parameters were extracted using custom-written Matlab procedures.
